# Improving the molecular spin qubit performance in zirconium MOF composites by mechanochemical dilution and fullerene encapsulation†

**DOI:** 10.1039/d3sc03089j

**Published:** 2023-08-16

**Authors:** Lucija Vujević, Bahar Karadeniz, Nikola Cindro, Andraž Krajnc, Gregor Mali, Matjaž Mazaj, Stanislav M. Avdoshenko, Alexey A. Popov, Dijana Žilić, Krunoslav Užarević, Marina Kveder

**Affiliations:** a Ruđer Bošković Institute Bijenička cesta 54 10000 Zagreb Croatia bahar.karadeniz@irb.hr dijana.zilic@irb.hr krunoslav.uzarevic@irb.hr kveder@irb.hr; b Department of Chemistry, University of Zagreb 10000 Zagreb Croatia; c National Institute of Chemistry Hajdrihova 19 SI-1001 Ljubljana Slovenia; d Leibniz IFW Dresden Helmholtzstrasse 20 D-01069 Dresden Germany

## Abstract

Enlarging the quantum coherence times and gaining control over quantum effects in real systems are fundamental for developing quantum technologies. Molecular electron spin qubits are particularly promising candidates for realizing quantum information processing due to their modularity and tunability. Still, there is a constant search for tools to increase their quantum coherence times. Here we present how the mechanochemical introduction of active spin qubits in the form of 10% diluted copper(ii)-porphyrins in the diamagnetic PCN-223 and MOF-525 zirconium-MOF polymorph pair can be achieved. Furthermore, the encapsulation of fullerene during the MOF synthesis directs the process exclusively toward the rare PCN-223 framework with a controllable amount of fullerene in the framework channels. In addition to the templating role, the incorporation of fullerene increases the electron spin–lattice and phase-memory relaxation times, *T*_1_ and *T*_m_. Besides decreasing the amount of nuclear spin-bearing solvent guests in the non-activated qubit frameworks, the observed improved relaxation times can be rationalized by modulating the phonon density of states upon fullerene encapsulation.

## Abstract 2

Mechanochemical encapsulation of C_60_ fullerene leads to a change in selectivity in the topology outcome and a significant increase in coherence among spin-active centers embedded in a moderately porous zirconium PCN-223 porphyrinic framework.

## Graphical abstract

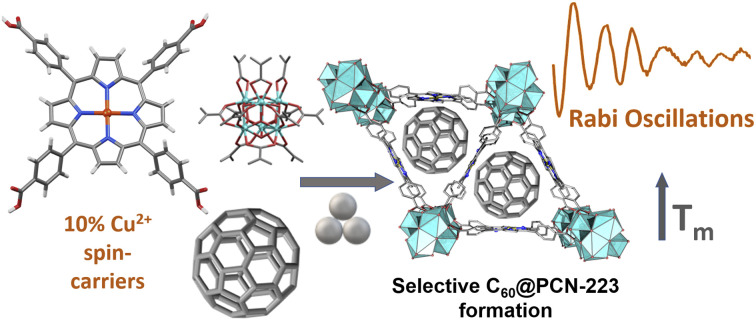

## 1 Introduction

A fundamental step towards developing of quantum technologies is building a qubit, a quantum bit that can exist in a coherent superposition state, unlike the classical bit that can possess only two states, 0 and 1.^1^ Among different ways of qubit realization, electron spin in molecular magnetic compounds, where the spin originates from the organic radical, transition metal ion or lanthanide, is particularly promising technology due to the facile manipulation of the electron quantum states by an electromagnetic irradiation.^2,3^ The main prerequisite for building a quantum device is the entanglement of qubits to achieve a state that is not the simple product of individual qubits. The inclusion of molecular spin qubits into the specifically tailored environment enables the study of the effect of spatial separation and interactions within the framework on the qubit performance, unraveling the role of phonon environment and spin–spin interactions on the operating speed of the qubit (spin–lattice relaxation time rate, 1/*T*_1_) and on the time limit in which the computation must be performed (phase-memory time, *T*_m_).^4^ Much effort is put into suppressing various origins of spin decoherence, such as due to the framework noise and interaction with nearby nuclei.^5^ To fully realize the potential of molecular spins for quantum information processing, it is necessary to build structurally well-defined arrays of spatially resolved molecular spins, hence combining improved spin coherence properties with optical or electrical access to their quantum states.^5–7^

Metal–organic frameworks (MOFs)^8,9^ assert as an ideal platform for controllable spatial resolution of qubit sites.^4,10^ These coordination polymers combine the coordination preferences of the metal cation nodes with different geometry of organic linkers to form a plethora of diverse topologies characterized by permanent porosity, different nature of the interior walls, and approachable metal nodes, not only on the surface but also in the interior of MOF particles. The modularity and tunability of MOFs, particularly MOFs of the fourth generation, made them one of the most investigated material classes today, with many potential application areas.^11,12^ In terms of quantum technologies, the variable topologies, periodicity, chemical diversity, and high internal ordering of MOFs provide an ideal and tunable platform for spatial resolution and manipulation of molecular spins, leading towards an emerging class of 3D-qubit array materials.^4^ Building such a MOF platform with long coherence time is a demanding task as the spin carriers need to be sufficiently separated from each other, while it is still not possible to maintain spatial precision at a molecular level in MOFs with highly diluted spin carriers. Also, MOFs inherently carry the magnetic noise of the ligands and guest molecules in the matrix, which may lead to the decoherence of qubits and usually shorter qubit lifetimes than required for quantum information processing (QIP) or quantum sensing.^13,14^ Most of these issues are, however, amendable, and it has been reported that including the copper(ii)-porphyrin building blocks in highly porous and activated MOFs results in spin coherence detectable up to liquid-nitrogen temperatures, making these materials attractive from the perspective of quantum information science.^4^ Therefore, understanding quantum system interactions with its respective environment plays a key step in controlling decoherence processes expressed in terms of *T*_1_ and *T*_m_ (an approximation of spin–spin relaxation time *T*_2_) relaxation times.^15^

**Fig. 1:**
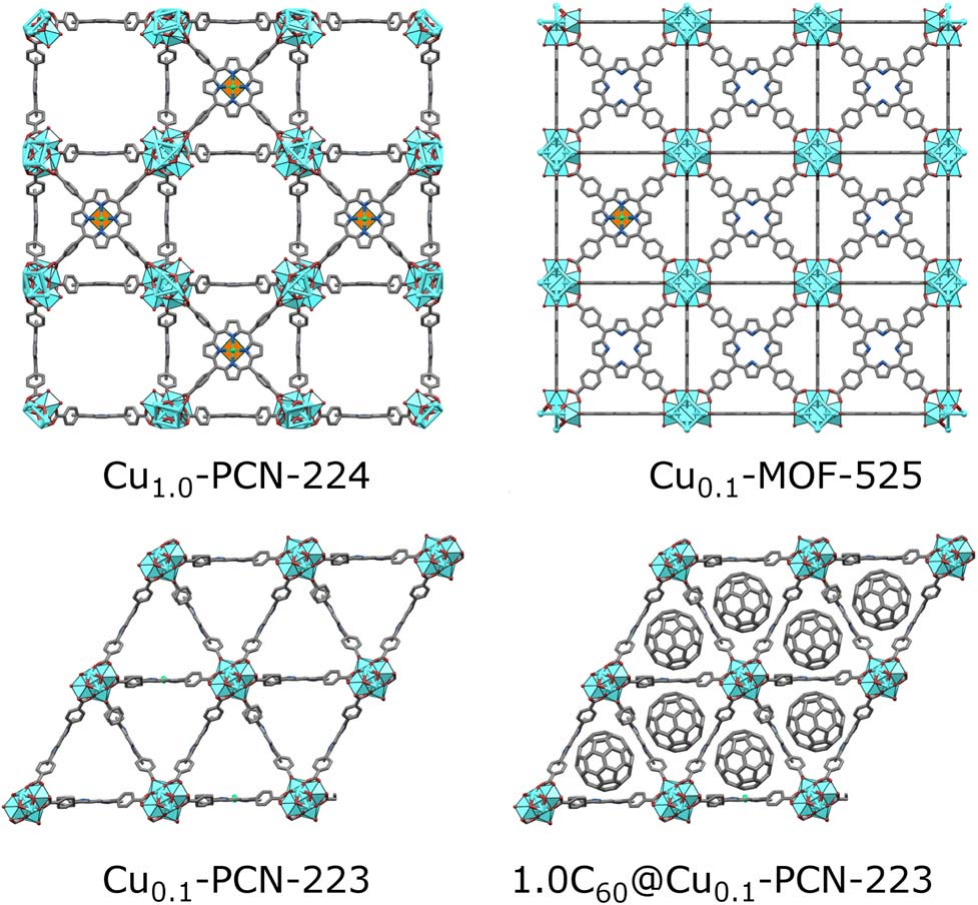
Porphyrinic Zr-MOFs considered for quantum information processing application; six-coordinated PCN-224 with a low population of linkers allowed for dense Cu(ii) spin-arrays (orange-green polyhedra in the center of porphyrin moiety).^4^ MOF-525 and PCN-223 involved in this study are twelve-coordinated, with a dense population of mixed TCPP linkers involving 10 percent of Cu(ii)-TCPP, which may lead to better stability of materials. The introduction of fullerene steers the formation exclusively towards the PCN-223 framework, with the fullerene populating the voids of the MOF.

A promising class of MOFs for quantum computing are porphyrinic zirconium carboxylate MOFs built from tetratopic porphyrinic ligand, tetrakis(4-carboxyphenyl)porphyrin (H_2_TCPP), and hexanuclear or octanuclear oxozirconium(iv) clusters.^16,17^ Zirconium carboxylate MOFs^18^ are widely researched due to their resistance to water and corrosive atmospheres,^19,20^ and framework-stability upon linker removal, leading to increased porosity of the material and larger distancing between the spin carriers.^21,22^ Despite zirconium(iv) clusters and resulting MOFs being diamagnetic, the spin active atom or group can be coordinated in the porphyrin center and efficiently separated by the coordination of the metalloporphyrin with binding carboxylate groups in the MOF structure.^23^ Porphyrin MOFs are rare zirconium MOFs displaying more than one topology.^24^ For some of the six known topologies, a polymorphic transition is also possible, for example, in the case of MOF-525 and PCN-223 polymorph pair (Fig. 1).^25^ Freedman's group, in their recent proof-of-concept work, exploited cobalt(ii) porphyrin molecular magnets diluted in a highly porous hexa-coordinated (on the zirconium cluster) PCN-224 MOF^17^ matrix for accomplishing a porous array of clocklike qubits, to tackle the issue with magnetic noise of the nuclear-spins rich MOF carrier.^26^ The same group showed how the incorporation of spin-active copper(ii) porphyrins into the PCN-224 results in porous molecular spin-based qubits with detectable coherence among the highly-concentrated spins in the framework up to 80 K (Fig. 1).^4,27^ In this context, copper(ii) porphyrins, where the copper bears *S* = 1/2 electronic spin, became attractive and extensively studied model systems for quantum information processing applications. The coherence parameters in terms of *T*_1_ and *T*_m_ were investigated using pulse ESR spectroscopy in addition to continuous wave (CW)-ESR.^10,23,28^ Not only the molecular spin-based qubits with high porosity and missing linkers, such as PCN-224, are suitable molecular spin qubit candidates. In our recent work, we showed how mechanochemistry^29^ provides a controllable pathway to synthesizing phase-pure twelve-coordinated PCN-223 and MOF-525 polymorphs.^25^ By introducing paramagnetic centers (Cu(ii), Fe(iii), and Mn(ii)) in the porphyrin linker, a superhyperfine splitting was observed in CW-ESR spectra of the more porous MOF-525 polymorph. In contrast, the same feature was less pronounced in PCN-223 where the nearest distance between the paramagnetic centers is 1.07 nm.^25^ The observed phenomenon denoted these materials as potential candidates for applications in quantum information processing.

An interesting strategy for manipulating the coherence among the spin carriers in framework materials is an introduction of guests that influence the framework properties and affect the height of the energy barrier, which may positively influence the *T*_1_-relaxation time.^30,31^ Our recent work showed that the introduction of C_60_ fullerene to sodalite ZIF-8 framework resulted in the stiffening of the framework and increased resistance to electromagnetic radiation, which depended on the ratio of the encapsulated fullerene.^32^ Here, we choose fullerene as an almost nuclear spin-free to reduce spin pollution, possibly increase the rigidity of the framework, and influence *T*_m_. The fullerene as a solid guest was also chosen to avoid a need for frozen CS_2_ suspensions as reported by Freedman's group^13^ or challenges related to the activation and removal of spin-pollutants in MOFs.^33^

Here, the presented study focuses on the modulation of copper(ii)-porphyrin qubit properties in the MOF-525 and PCN-223 polymorphic pair by diluting the spin carriers in the MOF structure and encapsulating the fullerene in the cavities of these spin-active frameworks. Whereas the proper distancing of the spin centers in MOFs is a well-known prerequisite for establishing coherence among the molecular spin carriers, where the MOF-525 showed to be advantageous, we are particularly interested to see how the controllable incorporation^32^ of a nanosized functional guest such as C_60_ fullerene within the channels of formed molecular spin-based qubit will be reflected on the coherence of the spin arrays in the robust and non-activated MOF samples.^34^

While previously MOF-525 was the predominant product of solution and mechanochemical syntheses,^25^ the introduction of fullerene inverts the selectivity of framework formation towards rarer PCN-223 polymorph, with the fullerene serving as a template and residing in triangular channels along the crystallographic *c* axis (Fig. S2†). For this study, we also prepared two fulleretic molecular spin-based qubits PCN-223 frameworks with different amounts of encapsulated C_60_ fullerene. The CW-ESR spectra analyses unravel magneto-structural characteristics of the copper-porphyrin qubit environment, including the local symmetry of the copper center in terms of *g* and hyperfine *A* tensors for both diluted molecular spin-based qubits. Besides direct measuring of spin-relaxation times, other pulsed ESR experiments were performed, such as HYSCORE and Rabi oscillations, to gain relevant information about the interactions with the environment's nuclei. The experimental and theoretical results point towards the multifunctional role of the fullerene for the prolongation of coherence times among the spin carriers, involving the reduction of spin noise and altering the spin–phonon interactions.

## 2 Results and discussion

Synthesis, DFT, IR, solid-state NMR, and CW/pulse ESR studies of 12-connected porpyhrinic zirconium MOF polymorphs, Cu-MOF-525 (cubic, ftw), Cu-PCN-223 (hexagonal, shp) and their composites, are presented. The copper(ii) ratio in the porphyrin centers of the synthesized MOFs was diluted to 10% copper(ii) as the copper(ii)–copper(ii) interactions cause line broadening in CW-ESR and destroy electron spin coherence in pulse ESR.

### 2.1 Synthesis

Our group recently reported the synthesis and controlled topology transformation of metallated and metal-free MOF-525 and PCN-223 by mechanochemistry.^25^ The here investigated multivariate Cu_0.1_-MOF-525 and Cu_0.1_-PCN-223 frameworks were synthesized in the same manner, with the difference that the CuTCPP and H_2_TCPP ligands were milled together in the desired ratio before the introduction of the zirconium source and the MOF formation, to ensure the homogeneous distribution of the diluted CuTCPP in the framework. The mechanochemical formation reactions are almost quantitative, yielding the insoluble MOF product without other H_2_TCPP byproducts observable in the PXRD of the crude product. The washing of the product is colorless, which further points towards the high efficiency of these reactions (the H_2_TCPP gives highly colored solutions even in low concentrations). A controlled amount of fullerene C_60_ (20 and 100 mol%) was successfully encapsulated into Cu_0.1_-PCN-223 using the one-pot liquid-assisted grinding (LAG) MOF formation.^32^ Noteworthy, in our previous work, the MOF-525 was predominant product, and PCN-223 polymorph could be obtained only in specific reaction conditions.^25^

Interestingly, the presence of C_60_ in the reaction mixture drives the formation exclusively towards hexagonal shp-C_60_@Cu_0.1_-PCN-223 polymorph, even with the reaction conditions that would in all cases afford the cubic ftw-Cu_0.1_-MOF-525 phase.^25^ C_60_ thus acted as a template providing the C_60_@Cu_0.1_-PCN-223 composite, and no traces of MOF-525 have been detected in any product. Tentatively, the arrangement of the linkers and channel size of PCN-223 polymorph is more suitable for encapsulating C_60_. While the similar templating effect of C_60_ has been reported previously on tuning the cavity size of multi-porphyrin macrocycles,^35^ no similar effect has been, to the best of our knowledge, reported for any zirconium carboxylate framework. The washing of the crude milling product was almost colorless, indicating, together with the FTIR and PXRD analyses, that the C_60_ guest was included in the pores of the frameworks in its entirety. After the C_60_ capture, DMF is still present in the pores of the products, but in a lower amount than in the non-fulleretic analogs (Fig. S3†). The PCN-223 structure has voids along the crystallographic *a*-axis that are too narrow for the accommodation of C_60_, but suitable for smaller DMF molecules (Fig. S2†).

**Fig. 2:**
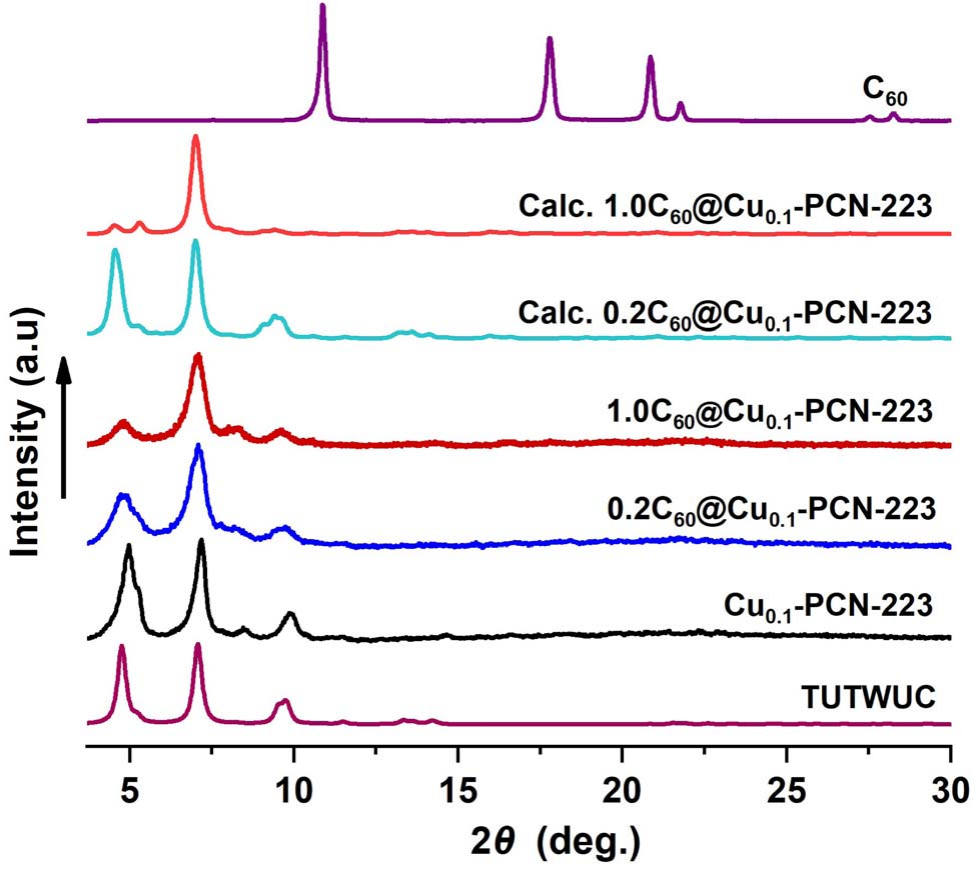
PXRD data for Cu_0.1_-PCN-223 and the C_60_@Cu_0.1_-PCN-223 composites *vs.* PXRD data for PCN-223 simulated from the single crystal diffraction data (Cambridge Crystallographic Data Centre (CCDC) code: TUTWUC) and two fulleretic composites optimized in this study (please see Section 2.2).

Synthesized MOFs and MOF composites are phase-pure and highly crystalline, and no free fullerene is visible in products after washing (Fig. 2). The disappearance of C_60_ peaks in the PXRD patterns confirms the absence of C_60_ crystals on the surface of MOF composites. PXRD data also provide a qualitative measure of the encapsulation of C_60_ within the PCN-223 framework. The intensity of the low angle peak, 2*θ* = 4.80, decreases due to the modified electron density in the MOF cage as a result of loading C_60_ in the channels of PCN-223 framework,^32,36^ which is also corroborated here by DFT. Calculated PXRD data closely matched the experimentally observed PXRD data, supporting the reduction of the low-angle peak intensity (Fig. 2), which is the effect of guest inclusion into the cage, similar to the previously observed behavior in fulleretic ZIF-8 hybrids.^32^ The weak Bragg reflection occurring in the fulleretic MOFs derived from the DFT study (please see Section 2.2) and not clearly visible in experimental data is likely due to the fullerene being fixed in one position, while the spectroscopic and computational studies suggest it to be heavily rotationally disordered.

Due to hard-to-control defects in the MOF structures and the dynamic nature of the MOF pore content that can exchange with the atmosphere, the analysis of MOFs composition is a demanding task, which is, for example, best visible in the problems related to the determination of one of the most researched and essential features of MOFs, their porosity.^37^ Zirconia porphyrin MOFs are not an exemption, showing up to 40% of missing linker defects without the collapse of the MOF structure.^38^ For the samples prepared in this work, the elemental analysis showed missing linkers defects in all products, an increase in carbon content, and a decrease in the hydrogen and nitrogen content after the encapsulation of fullerene. Here however, we primarily aimed to determine the ratio of copper to zirconium to establish whether the targeted implementation of the 10% of copper(ii) spin carriers to the framework was successful since the variation in copper(ii) content can affect the *T*_m_. To quantify the Cu/Zr ratio in the studied multivariate MOFs and establish the C_60_ content in their pores, we performed inductively coupled plasma mass spectrometry (ICP-MS) and energy dispersive X-ray spectroscopy on a Cs-probe corrected transmission electron microscope (EDAX-TEM) analyses (Fig. S1†).

ICP-MS data found the molar ratio of Zr(iv) : Cu(ii) to be 18 : 1 for C_60_@Cu_0.1_-PCN-223 (20 : 1 estimated), 1.92 : 1 for C_60_@Cu_1.0_-PCN-223 (2 : 1 estimated), and 20 : 1 for fullerene-free Cu_0.1_-MOF-525 (20 : 1 estimated). The analysis showed *ca.* 10-fold lower quantity of Cu(ii) spin carriers in the framework with diluted Cu(ii) spin carriers, C_60_@Cu_0.1_-PCN-223. Also, the absolute values of Zr(iv) and Cu(ii) in the fulleretic frameworks confirmed the high loading of C_60_ in the framework, as can be seen by the differences in the Zr(iv) and Cu(ii) content established for C_60_ encapsulated and C_60_-free MOF frameworks (ESI, Materials and methods†). The deviations from the idealized formulae may be due to the defects in structure and variable content of guests in the framework that we could not remove from the framework by standard activation procedures. EDAX-TEM showed homogeneous distribution of copper(ii) spin carriers in both fulleretic phases, the C_60_@Cu_0.1_-PCN-223 and C_60_@Cu_1.0_-PCN-223 (ESI, Fig. S1†). The relative molar ratio of the Zr/Cu in these samples corresponds well to the ICP-MS analysis, being approximately ten times higher for C_60_@Cu_0.1_-PCN-223 than the Zr/Cu for C_60_@Cu_1.0_-PCN-223. The absolute values and the carbon content in the frameworks were not possible to estimate by this method due to the samples being collected at the carbon foil influencing the measurement.

We can conclude from these analyses that the mechanochemical formation provided MOFs with the desired ratio of Cu-spin carriers in the framework and the homogeneous distribution at the TEM resolution. It must be noted that we could not completely control the distribution of the CuTCPP in the framework at a molecular level. As in other materials with diluted active spin carriers (for example, defects in nano-diamonds), the controllable implementation of diluted active species to MOFs is still a complex and hard-to-achieve issue. It is possible in some specific cases, for example, when the ligands have different binding functionalities, as in the case of bimetallic MOF-74 formation.^29,39^ Here, however, Cu-TCPP and H_2_TCPP have the same binding functionalities and affinity towards zirconium-MOF formation, so their implementation to the framework is largely stochastic. Regardless, the ESR analyses showed efficient separation of copper centers in the formed multivariate MOFs (please see section CW-ESR spectroscopy below).

### 2.2 DFT study

The system size allows a comprehensive DFT study (DFT/PAW/PBE-D, see ESI† for computational details) of the stability and dynamics of the PCN-223 framework with C_60_ molecules inside. The PCN-223 framework is relatively spacious, and after full optimization, no significant change in the C–C bond parameters of the C_60_ fullerene itself could be observed. The calculations also reveal a lack of significant affinity of the fullerene molecules toward the MOF framework, and the C_60_ molecules tend to stay in the middle of the voids.

**Fig. 3:**
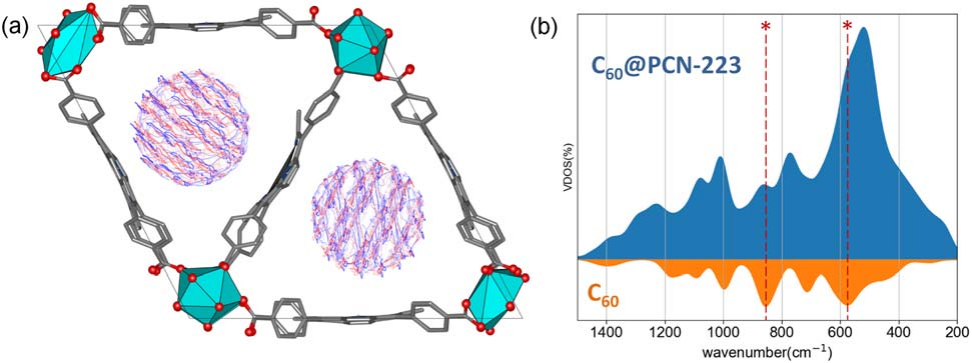
(a) Superposition of time-averaged snapshots for C_60_ within a 100 ps time-window of C_60_@PCN-223 dynamics at DFTB level of theory. (b) MD recovered the total and projected vibrational density of states for C_60_@PCN-223 and C_60_, where asterisks mark the most profound contribution by C_60_ in the total VDOS.

Within the channel, molecules appear to be well isolated from other parallel channels, although the distance between the closest molecules from the adjacent channels is roughly 6 Å (Fig. S2†). At the same time, the distance between the C_60_ molecules within a single channel is 8 Å, which undermines the possibility of significant electronic interaction between them. High-density fillings would be required to provide significant intermolecular interaction among the fullerenes within the channel. Further investigation of the system using the DFTB/MD method reveals that molecules can freely rotate on-site (Fig. 3a). Fig. 3a shows the time-averaged trajectory of the carbon cage atoms along the 100 picosecond trajectory while the vibrational densities of states are shown in Fig. 3b. The time-averaging smooths out continuous molecular motion along with the principal components of rotations. Within the channel, such a rotation would undoubtedly lead to a sizeable disorder. The aforementioned dense packing might partially prevent intense rotations.

### 2.3 FTIR-ATR spectroscopy

FTIR-ATR data of Cu_0.1_-MOF-525, Cu_0.1_-PCN-223, and (20% and 100%), C_60_@Cu_0.1_-PCN-223 are consistent with their counterparts in the literature.^25^ The appearance of a new vibrational peak at 526 cm^−1^ with a slight shift in the spectrum of (20% and 100%) C_60_@Cu_0.1_-PCN-223 is clearly assigned to the most dominant vibration signal of pristine C_60_, at 523 cm^−1^, proving the incorporation of C_60_ into the framework while the other vibration peaks of pristine C_60_ overlap with the framework features (Fig. S3†). The FTIR data reveals the presence of formamide solvent in the framework.

### 2.4 NMR spectroscopy

**Fig. 4:**
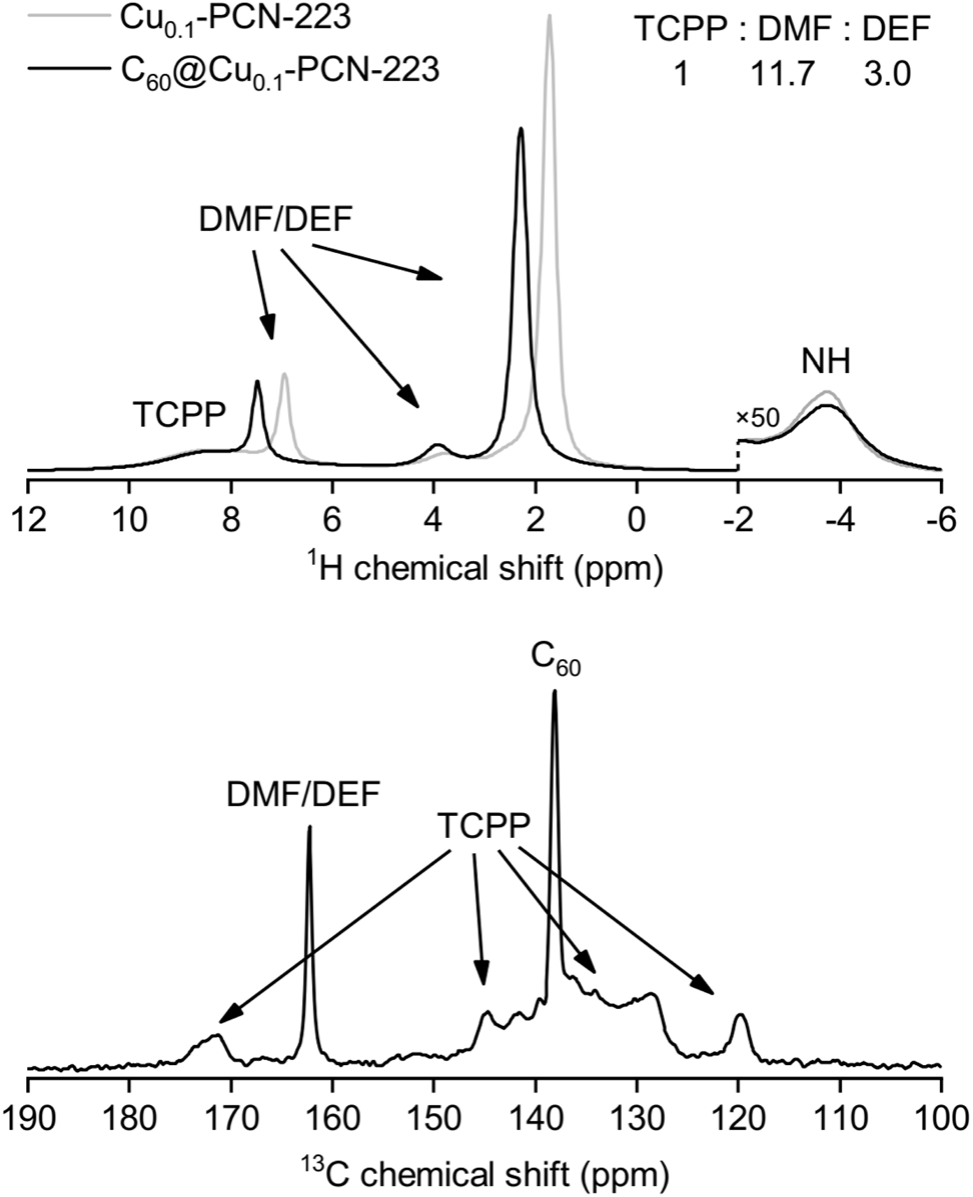
(Up) ^1^H MAS NMR spectra for Cu_0.1_-PCN-223 and 1.0C_60_@Cu_0.1_-PCN-223 at 300 K. (Down) ^13^C MAS NMR spectrum of 1.0C_60_@Cu_0.1_-PCN-223 at 300 K. The signals from dimethylformamide (DMF), diethylformamide (DEF), H_2_TCPP linker and fullerene (C_60_) are indicated on the spectra.

The results of solid-state NMR spectroscopy are presented in Fig. 4. Peak assignment was performed from the analysis of CPMAS and HETCOR spectra (Fig. S4 and S5†). The signal of ^13^C nuclei of C_60_ in the C_60_@Cu_0.1_-PCN-223, appearing at *ca.* 138 ppm, is narrow, suggesting that fullerene is free-standing and rotating inside the MOF channels. Any stronger anisotropic interaction with proximal paramagnetic copper centers is thus motionally averaged out. In the sodalite zeolitic-imidazolate-framework-8, fullerene was completely immobilized in the discrete voids of the framework, and its inclusion led to the stiffening of the framework.^32^ Here, the ^13^C MAS NMR spectra of C_60_@Cu_0.1_-PCN-223 in the region between 125 ppm and 135 ppm, and at about 145 ppm show that the incorporation of C_60_ to the PCN-223 framework leads to small shifts in the TCPP signal position, but it is hard to determine the precise effect of the C_60_ inclusion on the framework properties. ^1^H and ^13^C MAS recorded for 1.0C_60_@Cu_1.0_-PCN-223 corroborate that the DMF and DEF used for washing and aging are still present within the pores of the network. Moreover, the downfield shifts of the ^1^H signals suggest that the formamide molecules are either brought closer to the porphyrin rings upon C_60_ encapsulation or experience a reduced chemical shift averaging effect due to their motion being impeded by the encapsulated fullerenes.^40^

### 2.5 CW-ESR spectroscopy

**Fig. 5:**
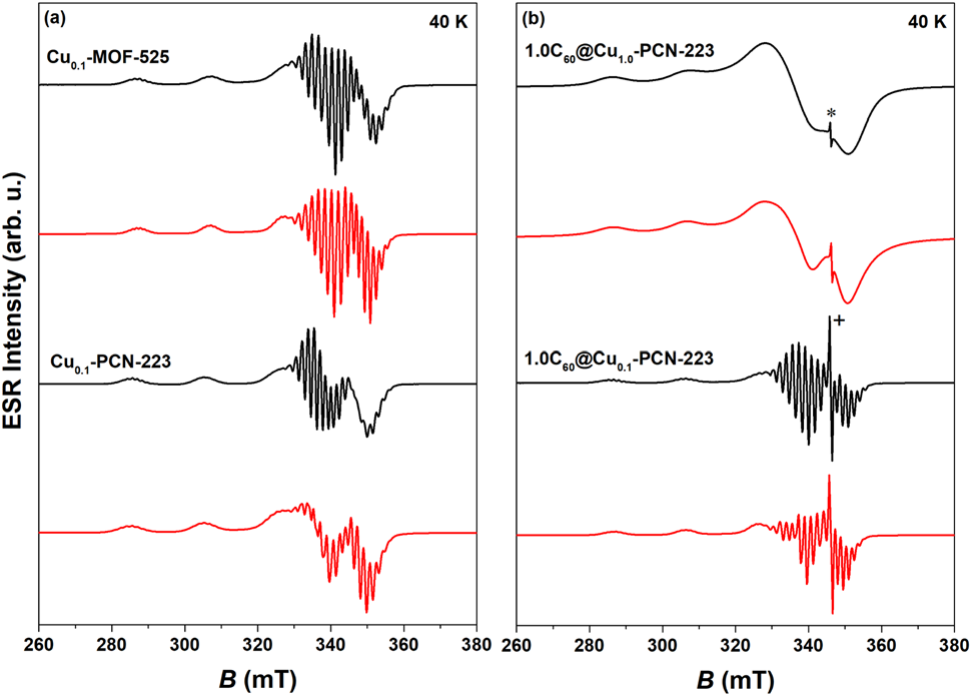
Experimental (black) and simulated CW-ESR spectra (red) of various MOFs recorded at 40 K: (a) Cu_0.1_-MOF-525 and Cu_0.1_-PCN-223, (b) 1.0C_60_@Cu_1.0_-PCN-223 and 1.0C_60_@Cu_0.1_-PCN-223. Asterisk in the figure indicates the fullerene-radical (FR) signal at *g* ≈ 2.003 while “+” denotes truncation of the peak. Parameters of the simulated spectra are reported in Table S1.†

CW-ESR spectra of various MOFs recorded at 40 K showing axial symmetry are presented in Fig. 5. It can be noticed that both Cu_0.1_-MOF-525 and Cu_0.1_-PCN-223 exhibit typical CW-ESR spectra of the diluted copper porphyrin monomers (Fig. 5a).^28,41^ The strong hyperfine coupling of the unpaired electron spin with the copper nuclear magnetic moment (*I* = 3/2) results in four lines, three of which are resolved in the low-field part of the spectrum. In comparison with the previously reported ESR spectra, which did not reflect the interaction with four nitrogen atoms from the porphyrin macrocycle in the first derivation of the absorption ESR spectra due to the high copper concentration (100% Cu, Cu_1.0_-PCN-223),^25^ here presented data exhibit strong modulation of the high-field part of the spectrum (Fig. 5a). This superhyperfine interaction in the ESR spectra of a sample with a lower concentration of copper ions (10% Cu) provides evidence of the capability of mechanochemical procedures to dilute metal ions in the molecular framework in a largely controllable manner.

The experimental spectra were simulated using EasySpin software^42^ assuming axial symmetry of **g** tensor and **A** hyperfine tensor while all four nitrogen atoms in the porphyrin ring were assumed to be equivalent.^43^ In specific, the tensor components *g*_‖_ and *A*_‖_ correspond to the orientation perpendicular to the porphyrin plane while *g*_⊥_ and *A*_⊥_ represent planar, plane orientation.^4^ In the interaction of the copper electron spin (*S* = 1/2) with the copper nucleus spin (*I*_Cu_ = 3/2) the same hyperfine coupling for natural-abundance mixture of copper isotopes (^63^Cu and ^65^Cu) was assumed.^41^ Therefore, the following spin-Hamiltonian for copper electron spin was used:

1 *H*_Cu_ = *μ*_B_*BgS* + *SA*_Cu_*I*_Cu_ + *SA*_N_*I*_N_

where *B* is external magnetic field while the constant *μ*_B_ is Bohr magneton. The parameters obtained from the simulations are given in Table S1† and are consistent with the data reported in the literature.^4,44^ In specific, the spectra exhibit *g*_‖_ > *g*_⊥_ > *g*_e_ indicative of the d_*x*^2^–*y*^2^_ copper electron spin ground state. No essential difference of **g**-tensor values in Cu_0.1_-PCN-223 and Cu_0.1_-MOF-525 indicates no difference in their respective orbital angular momentum contributions. Similarly, hyperfine/superhyperfine tensor differences are within the experimental variability of data. In addition, the tetrahedral distortion of the square planar copper geometry^45^ estimated from the ratio *f* = *g*_‖_/*A*^Cu^_‖_ indicates perfect square-planar geometry which pertains upon the incorporation of fullerene (Table S2†). The corresponding CW-ESR spectra of 1.0C_60_@Cu_1.0_-PCN-223 and 1.0C_60_@Cu_0.1_-PCN-223 are presented in Fig. 5b. Fullerene has a weak ESR signal (FR) at *g*_FR_ ≈ 2.002 due to defects in fullerene structure (Fig. S21† in ESI in ref. 32). This signal is even visible when the structure was loaded with 100% of Cu although the superhyperfine lines could not be resolved due to strong spin–spin interactions between copper cations (Fig. 5b). Additionally, there is a signal of empty H_2_TCPP (without copper ions in porphyrin rings) at a similar *g*-value as the fullerene-radical signal but this signal is broader, weaker, and suppressed by the stronger FR signals. Due to this fact, the simulation of the experimental spectra was done in terms of a mixture of spin species by combining the spin-Hamiltonian of copper given by eqn (1) and spin-Hamiltonian of FR while assuming *S*_FR_ = 1/2: *H*_FR_ = *μ*_B_*Bg*_FR_*S*_FR_. As C_60_ is known to be a good electron acceptor, and porphyrins are also electroactive, an electron transfer between the fullerene and porphyrin linkers in MOFs may occur. However, based on their respective redox potential, this possibility is unlikely.^46,47^

The simulation parameters^42^ and the corresponding spectra are given in Table S1† and Fig. 5b. The obtained data are in accordance with the NMR and DFT studies and the literature stating that encapsulated fullerene is free-standing inside the cage.^32^

### 2.6 Pulse ESR spectroscopy

**Fig. 6:**
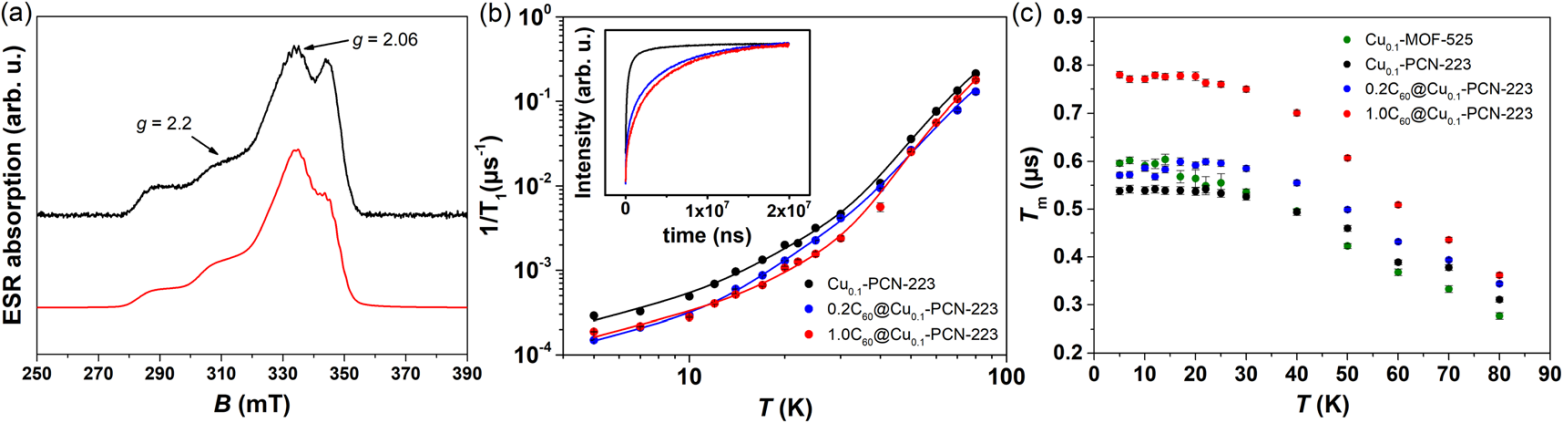
(a) Experimental (black) and simulated (red) two-pulse echo detected field-swept ESR spectrum of Cu_0.1_-PCN-223 recorded at *T* = 80 K. The tensor values *g*_‖_ = 2.2 and *g*_⊥_ = 2.06 labeled by arrows indicate the magnetic field spectral positions at which spin dynamics was undertaken. (b) Temperature dependence of the electron spin–lattice relaxation rate, 1/*T*_1_, of Cu_0.1_-PCN-223 (black), 0.2C_60_@Cu_0.1_-PCN-223 (blue) and 1.0C_60_@Cu_0.1_-PCN-223 (red) measured at *g*_‖_ spectral position. Full lines denote best-fits of the experimental data according to the eqn (2) with the parameters given in Table 1. In the inset to the figure, magnetization recovery curves are given in the inversion recovery experiment detected at 5 K. (c) Temperature dependence of the electron-spin phase-memory relaxation time, *T*_m_, in various MOFs at *g*_‖_ spectral position.

Two-pulse echo detected field-swept ESR spectra of various MOFs were recorded at 80 K in order to assign the spectral positions for relaxation time measurements in pulsed ESR experiments (Fig. 6a). Two Cu(ii) magnetic field resonances were chosen, which correspond to two principles **g**-tensor values *g*_‖_ = 2.2 and *g*_⊥_ = 2.06.^4^

#### 2.6.1 Spin–lattice relaxation time

The electron spin–lattice relaxation times, *T*_1_, of four MOF-samples: Cu_0.1_-PCN-223, 0.2C_60_@Cu_0.1_-PCN-223, 1.0C_60_@Cu_0.1_-PCN-223 as well as of Cu_0.1_-MOF-525 were measured across temperature range of 5–80 K (Fig. S7†). The shortest *T*_1_ was detected for Cu_0.1_-PCN-223, which, upon loading of the fullerene molecules into the structure, presented much longer *T*_1_ values (Fig. 6b). This phenomenon can be noticed in the whole temperature range studied. In specific, the longest *T*_1_ of *ca.* 6.7 (5.3) ms was measured at 5 K and *g*_‖_ position for 0.2C_60_@Cu_0.1_-PCN-223 (1.0C_60_@Cu_0.1_-PCN-223) as compared with the empty Cu_0.1_-PCN-223 which exhibited *T*_1_ of *ca.* 3.5 ms. After heating the samples to 80 K, *T*_1_ values decreased by three orders of magnitude and converged to similar *T*_1_ values in the range of 5 μs (Fig. 6b). For all the samples studied, *T*_1_ measured at *g*_‖_ was longer than the one at *g*_⊥_ position by a factor of two at the lowest temperatures studied (Fig. S7†). Interestingly, at the lowest temperatures studied *T*_1_ values of MOF-525 were similar to the values of PCN-223 loaded with fullerene while approaching the similar values of other MOFs upon heating up the sample (Fig. S7†). *T*_1_ data obtained here show one order-of-magnitude longer values at the lowest temperatures compared with data for copper-porphyrin qubits in Cu_0.1_-PCN-224 (ref. 4) and Zr-Cu-NU-1102 (ref. 27) while has the same order-of-magnitude values compared to the data for copper-porphyrin qubits in 2D nanosheets.^23^

The fullerene encapsulation alters the phonon density of states of the material, affecting thus relaxation times. To understand the experimental data, various mechanisms transferring the spin excitation energy to the crystal lattice were considered. Since no thermally accessible electronic excited states are expected for copper(ii) complexes, the Orbach-Aminov mechanism was neglected.^48,49^ Finally, three main processes of the spin–lattice relaxation rate, 1/*T*_1_, were assigned to adequately reproduce the experimental data: direct one-phonon, two-phonon Raman, and local mode-mediated processes.^48^ In this context, assuming the Debye type of phonon spectrum, experimental 1/*T*_1_ values were fitted according to equation^48,50^2
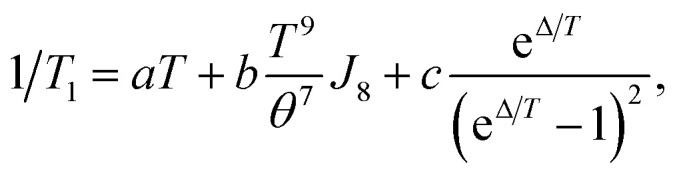
as shown in Fig. 6b while the best-fit parameters are given in Table 1 and with more statistical details in Table S3.† It should be stressed that due to the problem of reaching the global minimum in non-linear fitting with 5 unknown parameters, the obtained values here should be considered as an estimation of the possible parameters rather than the unique solutions. The first term describes the one-phonon process, and it is important at the lowest temperatures studied when the phonons with high energy are still not excited. The second term is related to the two-phonon Raman process with *J*_8_ denoting the transport integral, which was solved numerically, while *θ* is Debye temperature. The last term describes local-mode mediated process of energy *Δ*.^48,51^ The example of various terms contribution is illustrated in Fig. S8.†

**Table 1:** The best-fit parameters to the experimental spin–lattice relaxation rate data according to eqn (2) with a numerical evaluation of the transport integral and referring to Cu_0.1_-PCN-223 (without fullerene), 0.2C_60_@Cu_0.1_-PCN-223 and 1.0C_60_@Cu_0.1_-PCN-223 (with fullerene). *R*^2^ provides information about the goodness of fit of a model

Compound	*g*_position_	*a*, 1/(Kμs)	*b*, 1/(K^2^μs)	*θ*, K	*c*, 1/μs	*Δ*, K	*R* ^2^
Cu_0.1_-MOF-525	*g*_‖_	(3.2 ± 0.3)10^−5^	(4 ± 2)10^−5^	77 ± 22	7 ± 4	285 ± 35	0.999683
Cu_0.1_-MOF-525	*g*_⊥_	(6.±1)10^−5^	(4 ± 2)10^−5^	66 ± 35	9 ± 4	292 ± 31	0.999429
Cu_0.1_-PCN-223	*g*_‖_	(5.1 ± 0.3)10^−5^	(3 ± 1)10^−5^	74 ± 18	5 ± 2	273 ± 26	0.99984
Cu_0.1_-PCN-223	*g*_⊥_	(8.6 ± 0.7)10^−5^	(4.0 ± 0.9)10^−5^	67 ± 19	7 ± 2	290 ± 20	0.99979
0.2C_60_@Cu_0.1_-PCN-223	*g*_‖_	(2.9 ± 0.2)10^−5^	(3.3 ± 0.7)10^−5^	82 ± 12	4 ± 2	281 ± 36	0.999908
0.2C_60_@Cu_0.1_-PCN-223	*g*_⊥_	(6.1 ± 0.9)10^−5^	(3 ± 1)10^−5^	77 ± 36	5 ± 2	280 ± 33	0.999775
1.0C_60_@Cu_0.1_-PCN-223	*g*_‖_	(3.2 ± 0.2)10^−5^	(2 ± 1)10^−5^	94 ± 40	6 ± 2	289 ± 30	0.999683
1.0C_60_@Cu_0.1_-PCN-223	*g*_⊥_	(6.1 ± 0.5)10^−5^	(2 ± 2)10^−5^	94 ± 44	8 ± 3	306 ± 28	0.999718

The results of the fitting presented in Table 1 clearly show that the anisotropy of *T*_1_ regarding the spectral positions of *g*_‖_ and *g*_⊥_ is present in all the samples studied. The analogous anisotropy of *T*_1_ data was reported for similar copper porphyrin structures in PCN-224.^4,27^ Along with the seminal work presented by Eaton's group,^52^ the very recent work by Kazmierczak and Hadt^53^ has investigated both experimentally and theoretically a library of Cu(ii) and V(iv) complexes exhibiting comparable *T*_1_ anisotropy. A theory based on the spin–orbit coupling wave functions and different ligand field contributions at two *g*-positions successfully reproduced inversion recovery measurements performed at 100 K. It is interesting to note that here observed anisotropy of *T*_1_ (Table 1) is most visible in the region of lowest temperatures studied where the direct process is governing the electron spin relaxation.

Additionally, Table 1 shows two main differences between MOF samples with and without fullerene. First, the samples containing fullerene exhibit a smaller value of parameter *a* than those without fullerene, implying a slowing down of the direct process. Second, related to the Raman process, a considerable trend in Debye temperature towards higher values in the presence of fullerene can be noticed despite large uncertainties of the fitted values. The larger Debye temperature might be related to the combined effect of the increased number of oscillators and the speed of sound upon fullerene incorporation in MOF matrix.^54^ The values for Debye temperatures obtained here have the same order of magnitude as the value of 102 K found for MOF-5.^55^ Regarding the local-mode mediated process, energy values of *Δ* ≈ 280 K (200 cm^−1^) were obtained (Table 1). This value is too high for the acoustic phonons to be involved, while it is too low to account for the lowest fullerene vibration (270 cm^−1^). Therefore, we can assume that these modes probably belong to MOF since a similar *Δ* was found for all the samples irrespective of the incorporation of fullerene. Therefore, we can propose that fullerene encapsulation prolongates *T*_1_ relaxation time of copper porphyrin MOFs by slowing down direct and Raman processes.

#### 2.6.2 Phase-memory relaxation time

The temperature dependence of the electron-spin phase-memory relaxation times, *T*_m_, of various MOFs is presented in Fig. 6c and S9† for *g*_‖_ spectral position. At 5 K, the longest *T*_m_ value around 800 ns was detected in 1.0C_60_@Cu_0.1_-PCN-223. For comparison, the shortest *T*_m_ was observed in Cu_0.1_-PCN-223 (540 ns) whereas Cu_0.1_-MOF-525 featured *T*_m_ of 600 ns (Fig. 6c). Therefore, *T*_m_ is affected by the content of fullerene in the structure giving rise to larger values. The improved values can be ascribed to the partial exclusion of nuclear spin-bearing solvent by the almost nuclear spin-free fullerene guests. Namely, ^13^C isotope with nuclear spin *I* = 1/2 has a natural abundance of 1.07%. The obtained values are of the same order of magnitude as for Cu_0.1_-PCN-224 (ref. 4) and for copper-porphyrin qubits in 2D nanosheets^23^ while they are the order-of-magnitude larger compared to the values obtained for Zr-Cu-NU-1102.^27^ Upon increasing the temperature above 5 K, *T*_m_ exhibited no temperature dependence up to *ca.* 30 K. Here, spectral diffusion as a temperature-independent process can be assumed. Further heating of the samples (40–80 K) revealed a gradual decrease of *T*_m_ according to the Arrhenius type of thermally activated processes with the larger activation energy when the fullerene is present in the MOF structure (Fig. S9†). According to the thorough work of Hadt's group,^51,56^ the extensive theoretical work, including ligand field theory and mode-by-mode analyses of various molecular vibrations which might participate in spin decoherence, is required to assign specifically which thermally activated process is taking place, the task beyond here presented study.

#### 2.6.3 HYSCORE

**Fig. 7:**
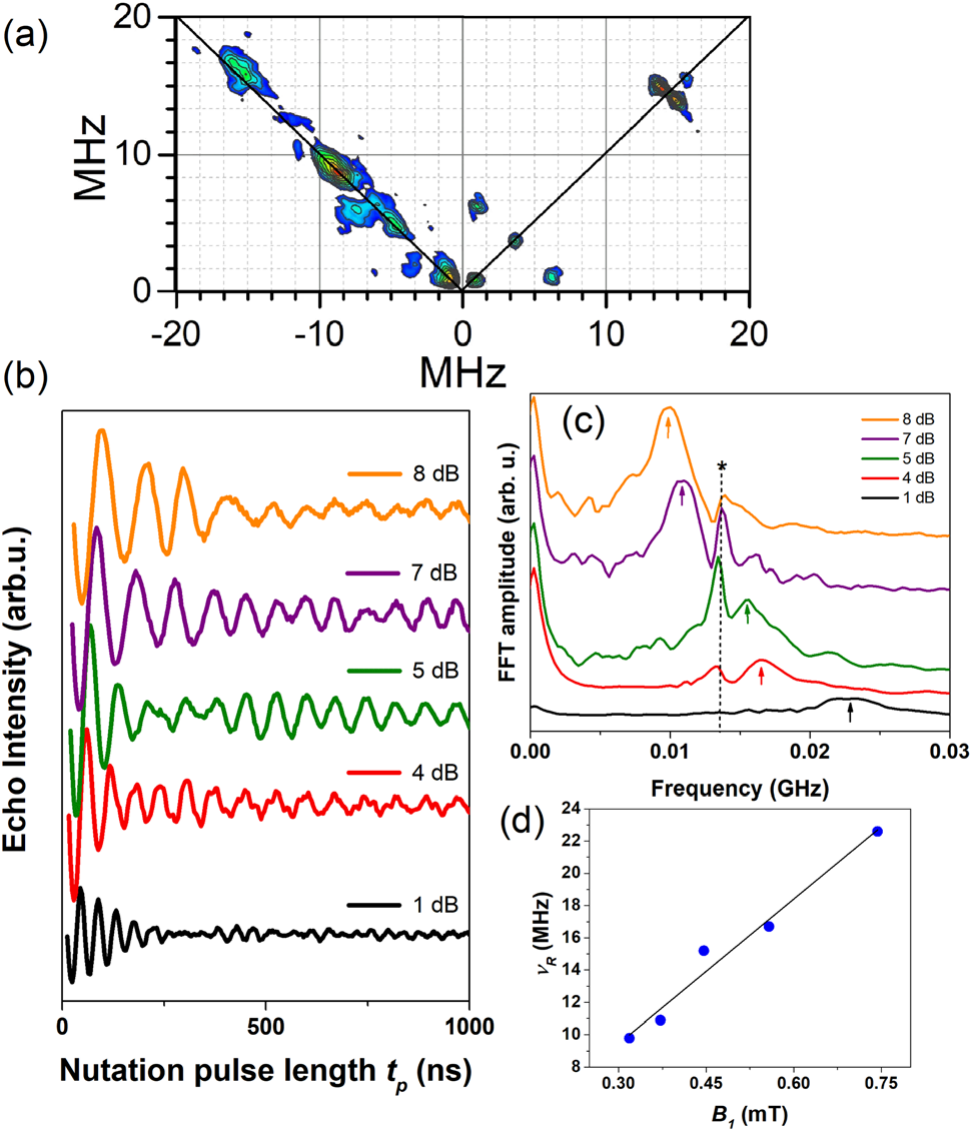
(a) HYSCORE performed at 5 K and *g*_‖_ spectral position for 1.0C_60_@Cu_0.1_-PCN-223 plotted after data processing. (b) Rabi oscillations recorded for 1.0C_60_@Cu_0.1_-PCN-223 at 20 K and *g*_‖_ spectral position applying various microwave power attenuation. (c) Fast Fourier transform (FFT) of the Rabi oscillations with an asterisk denoting Larmor frequency of proton spins ≈13.5 MHz while arrows denote Rabi frequencies. (d) Linear dependence of the Rabi frequency, *ν*_R_, as a function of the oscillating microwave field, *B*_1_, calculated according to ref. 63 and 64.

Two-pulse electron Hahn spin-echo decay curves of various MOFs presented strong modulation depths in the coherence time decays reflecting specific nuclear spin environment affecting the copper electron spin relaxation (Fig. S10†). These measurements are equivalent to two-pulse ESEEM experiments which can be extended to the three-pulse version and further to the two-dimensional level, HYSCORE. Therefore, to help the assignment of the observed modulation patterns, HYSCORE measurements were performed (Fig. 7a). In the (+,+) quadrant, one can note the signals from the weakly coupled nuclei^57,58^ appearing on the diagonal at *ca.* 0.84, 3.7 and 14.4 MHz. The latter peak can be assigned unambiguously to the X-band proton Larmor frequency and corresponds to the hyperfine interaction of copper electron with the remote hydrogen (^1^H) nuclei. The other peaks could be assigned to the electron interaction with ^14^N and/or ^13^C nuclei (Fig. S11†). Specifically, ^14^N with nuclear spin *I* = 1 exhibits non-spherical charge distribution requiring quadrupolar interaction to be considered. The low-frequency peak at 0.84 MHz is close to the cancellation limit when the hyperfine and nuclear Zeeman terms match in one of the electron spin manifolds giving rise to the ^14^N quadrupolar transitions.^59,60^ For weakly coupled nitrogen, the strongest cross-peaks in the HYSCORE spectrum generally correlate to double-quantum (DQ) frequencies.^57^ Therefore, a diagonal peak at 3.7 MHz might be assigned to the DQ transition of the solvent nitrogen.^57,60,61^ However, since ^13^C nuclei have Larmor frequency of 3.7 MHz at X-band, they can also contribute^28^ and the best way to resolve ^13^C from ^14^N would be to perform HYSCORE at higher frequency *e.g.* W-band what is beyond the scope of this work. Furthermore, ^14^N can exhibit 2DQ peak at *ca.* 9 MHz^62^ as can be noticed in the (−,+) quadrant in Fig. 7a. The assignments pointing to the interaction with nitrogen nuclei refer to remote nitrogens from the solvent (more than 0.4 nm away from the copper electron spin^60^) since directly coordinated ones from the porphyrin ring exhibit large hyperfine coupling which cannot be detected in the HYSCORE experiment but do affect CW-ESR spectra (Table S1†). As a support for this reasoning one should notice that NMR and FTIR spectra show signals from formamide additives even in 1.0C_60_@Cu_0.1_-PCN-223, thus, corroborating that formamide solvent molecules are still present in the cavities of PCN-223, likely those present along the crystallographic *a* axis.

#### 2.6.4 Rabi oscillations

To prove that the samples studied have the potential for application in the field of quantum information processing,^26^ the nutation experiments at different microwave powers and 20 K were performed at *g*_‖_ (Fig. 7b) and *g*_⊥_ (Fig. S11†) spectral positions. The interaction between the qubit and the applied microwave field is described by the Rabi frequency, which gives the rate of transition between the ground and excited states. To achieve coherent manipulation between the states, it is a prerequisite for a quantum information processing application to obtain Rabi oscillations with a frequency having linear dependence on the applied microwave field.^65,66^ After Fourier transformation of the time domain data, two important types of peaks can be noticed in Fig. 7c. One type is centered at *ca.* 13.5 MHz and shows variable intensity depending on the microwave power. Because these peaks do not present frequency shifts, they can be assigned to coherent electron-proton oscillations. In specific, their intensity reaches a maximum when the Larmor frequency of proton spins matches electron nutation frequency.^10^ The second type of peaks represent the nutation frequency peaks, *ν*_R_, as they gradually shift from *ca.* 9 to 23 MHz when the microwave magnetic field increases. It is important to note that these peaks exhibit approximately linear dependence on the microwave field *B*_1_, as seen in Fig. 7d. This result indicates the possibility of creating an arbitrary superposition of states, thus fulfilling the requirement for possible applications of these types of material in quantum information processing.^65,67^

## 3 Conclusion

In summary, this study shows how the mechanochemical processing accomplished through a robust one-pot ball-milling procedure allowed for control over the ratio of active spin copper(ii) carriers and the amount of fullerene C_60_ guest embedded into the channels of moderately porous porphyrinic zirconium MOFs with twelve-coordinated oxocluster nodes. The use of C_60_ leads to topological selectivity by governing the formation towards the hard-to-obtain PCN-223 phase, even from reactions that exclusively yield phase-pure cubic MOF-525 polymorph in the absence of fullerene. Here presented experimental evidence points to the possibility of fine-tuning the electron spin relaxation times *via* fullerene content in the MOF cavity, where an increase in *T*_1_ and *T*_m_, detected in MOF fulleretic hybrids, can be directly related to the ratio of the fullerene guest. It should be emphasized that the increase in the electron spin coherence was observed in the presence of solvent guests without the necessity for MOF activation which usually leads to the collapse of the structure. We propose that fullerene encapsulation prolongates both relaxation times of copper porphyrin MOFs, first through the partial exchange of the nuclear spin-bearing solvents with rigid almost nuclear spin-free fullerenes affecting *T*_m_, but also through slowing down direct and Raman processes affecting thus *T*_1_. The nutation experiments presenting linear dependence of the Rabi oscillation on the intensity of the oscillating field indicate the possibility of creating an arbitrary superposition of states, proving that coherent manipulation between the electron spin states in the studied fulleretic MOF hybrids can be achieved. In this way, even the non-activated and densely coordinated zirconium-porphyrin frameworks become viable molecular spin qubit matrices, where the inferior and least porous Cu_0.1_-PCN-223 candidate becomes the best qubit performer after the fullerene inclusion. Using similar solid-state strategies, we aim to test how altering the spin carrier species and including fullerene and endometallofullerene derivatives in more porous zirconium-porphyrinic MOFs, such as MOF-525 and well-established six-coordinated framework PCN-224, will affect their spin coherence properties.

## Data availability

All the supporting calculation and experimental data is a part of ESI.†

## Author contributions

The project was conceived and supervised by KU, BK, DŽ, and MK. BK prepared MOF materials and their diluted and composite phases. NC was involved in the synthesis and characterization of ligands. LV, DŽ, and MK performed CW and pulse ESR measurements and analyses. AK and GM conducted, analyzed, and prepared solid-state NMR spectroscopy. MM carried out and analysed energy dispersive X-ray spectroscopy (EDAX) measurements. SMA and AAP conducted DFT (density functional theory) calculations and molecular dynamics (MD) simulations and wrote part of the DFT study. The initial draft of the manuscript was written by DŽ, BK, MK, and KU. BK and LV prepared the figures and graphical abstract. All authors discussed the results and contributed to the final preparation of the manuscript.

## Conflicts of interest

There are no conflicts to declare.

## Supplementary Material

SC-014-D3SC03089J-s001
