# Supplementary material for: Improving the molecular spin qubit performance in zirconium MOF composites by mechanochemical dilution and fullerene encapsulation
Source: Chem Sci. 2023 Aug 16;14(35):9389–99. doi: 10.1039/d3sc03089j (PMC10498684; doi:10.1039/d3sc03089j)
Supplement: SC-014-D3SC03089J-s001 [file SC-014-D3SC03089J-s001.pdf]

Electronic Supplementary Material (ESI) for Chemical Science.
This journal is © The Royal Society of Chemistry 2023

Supplementary Information

for

Improving the molecular spin qubit performance in zirconium MOF composites by mechanochemical dilution and fullerene encapsulation

by

Lucija Vujević,[a] Bahar Karadeniz,*[a] Nikola Cindro,[b] Andraž Krajnc,[c] Gregor Mali,[c] Matjaž Mazaj,[c] Stanislav M. Avdoshenko,[d] Alexey A. Popov,[d] Dijana Žilić,*[a] Krunoslav Užarević,*[a] Marina Kveder*[a]

[a] Ruđer Bošković Institute, Bijenička cesta 54, 10000 Zagreb, Croatia.
[b] Department of Chemistry, University of Zagreb, 10000 Zagreb, Croatia.
[c] National Institute of Chemistry, Hajdrihova 19, SI-1001 Ljubljana, Slovenia.
[d] Leibniz IFW Dresden, Helmholtzstrasse 20, D-01069 Dresden, Germany.

E-mail: bahar.karadeniz@irb.hr, dijana.zilic@irb.hr, krunoslav.uzarevic@irb.hr, kveder@irb.hr

Contents

S1 Experimental **5**
S1.1 Synthesis 5
S1.2 Powder X-ray diffraction 7
S1.3 Fourier-transform infrared spectroscopy (FTIR) 7
S1.4 Inductively Coupled Plasma Mass Spectrometry (ICP-MS) 7
S1.5 Elemental analysis 7
S1.6 TEM microscopy 7
S1.7 Modeling 8
S1.8 Solid-state nuclear magnetic resonance (NMR) 8
S1.9 ESR spectroscopy 8

S2 Results and discussion **10**
S2.1 EDAX-TEM microscopy 10
S2.2 DFT 10
S2.3 Fourier-transform infrared spectroscopy (FTIR) 11
S2.4 Solid-state CPMAS and CP-HETCOR NMR spectroscopy 12
S2.5 CW ESR spectroscopy 14
S2.6 Pulse ESR spectroscopy 16

List of Figures

S1 Elemental mapping on the selected particles of the investigated materials - (a) C_{60}@$Cu_{0.1}$-PCN-223 sample with the corresponding distribution of (b) Zr and (c) Cu; (d) C_{60}@$Cu_{1.0}$-PCN-223 sample with the corresponding distribution of (e) Zr and (f) Cu. Estimated Zr/Cu molar ratios are 45 (33 wt. Zr and 0.5 wt. Cu) and 4.0 (17 wt. Zr and 3 wt.Cu) for C_{60}@$Cu_{0.1}$-PCN-223 and C_{60}@$Cu_{1.0}$-PCN-223 samples respectively. 10
S2 The view down the crystallographic a) *c*-axis and b) *a*-axis of the optimized structure of 1.0C_{60}@-PCN-223 showing the narrower channels suitable for the accommodation of DMF molecules. 10
S3 FTIR-ATR spectroscopy data for 1.0C_{60}@$Cu_{0.1}$-PCN-223, 0.2C_{60}@$Cu_{0.1}$-PCN-223, $Cu_{0.1}$-PCN-223, DMF, and C_{60}, respectively. The bands denoting the successful encapsulation of C_{60} are marked with an asterisk (*). The spectral distortion visible in the spectrum of the C_{60} can be ascribed to the Christiansen effect.[1] It is present in all the C_{60} spectra taken, no matter the mode of collection (ATR or transmission in a KBr pastille). 11

S4 The ^1H–^{13}C CPMAS NMR spectra recorded for $Cu_{0.1}$-PCN-223 and $1.0C_{60}@Cu_{0.1}$-PCN-223 samples at 300 K. The blue color represents the CPMAS spectrum recorded at a very short mixing time, τ_{cp}, of 50 μs which reveals the protonated ^{13}C signals of TCPP. The ^{13}C signal of C_{60} at ca. 138 ppm is indicated by the arrow. The TCPP peaks indicated by numbers follow the notation of ^1H–^{13}C CP-HETCOR experiments shown in Fig. S5. 12
S5 The ^1H–^{13}C CP-HETCOR NMR spectrum of $1.0C_{60}@Cu_{0.1}$-PCN-223 sample recorded at 300 K. The ^{13}C signal of C_{60} at ca. 138 ppm is indicated in the figure.The TCPP peaks indicated by numbers follow the notation of ^1H–^{13}C CPMAS experiments shown in Fig. S4. 13
S6 CW-ESR spectra of $0.2C_{60}@Cu_{0.1}$-PCN-223 at indicated temperatures. Peak-to-peak linewidth W_{pp} and g_{FR} of fullerene-radical (FR) due to the defects in fullerene structure are given. 14
S7 Temperature dependence of the electron spin-lattice relaxation rate, $1/T_1$, in various MOFs as indicated in the legend for two spectral positions: a) $g_{\|}$ and b) $g_{\perp}$. Full lines denote simulations of the experimental data according to the Eq. S1 with the parameters given in Table 1. 16
S8 Temperature dependence of the electron spin-lattice relaxation rate, $1/T_1$, in $Cu_{0.1}$-PCN-223 at $g_{\|}$ spectral position. Experimental data (symbols) were simulated (lines) according to the Eq. S1 with the parameters given in Table 1. Individual components of direct, Raman and local modes are presented as indicated in the legend. 18
S9 Temperature dependence of the electron spin phase-memory relaxation rate, $1/T_m$, in various MOFs at $g_{\|}$ spectral position. The best fits according to Arrhenius equation are indicated with full lines. In the temperature interval from 40–80 K the activation energies are: 35±5 K ($Cu_{0.1}$-PCN-223, black), 48±5 K ($0.2C_{60}@Cu_{0.1}$-PCN-223, blue), 67±8 K ($1.0C_{60}@Cu_{0.1}$-PCN-223, red) and 37±4 K ($Cu_{0.1}$-MOF-525, green). 19
S10 Hahn echo intensity decay curves detected at 5 K and $g_{\|}$ spectral position for $Cu_{0.1}$-PCN-223 (black), $1.0C_{60}@Cu_{0.1}$-PCN-223 (red) and $Cu_{0.1}$-MOF-525 (green). In the Inset to the Figure the Fourier transform of the time domain data is presented. . 20
S11 (a) Rabi oscillations recorded for $Cu_{0.1}$-PCN-223 at 20 K and $g_{\perp}$ spectral position applying various microwave power attenuation. (b) Fast Fourier transform (FFT) of the Rabi oscillations with an asterisk denoting Larmor frequency of proton spins ≈13.5 MHz while arrows denote Rabi frequencies. (c) Linear dependence of the Rabi frequency, ν_R, as a function of the oscillating microwave field, B_1, calculated according to refs. 2 and 3. 21
S12 Left: Asymmetric unit of $Cu_{1.0}$-PCN-223 (Zr-light blue, N-blue, O-red, C-gray, Cu-orange). Right: 1/3 part of the asymmetric unit with 8 different Cu–C distances indicated. 22

List of Tables

S1 Spin Hamiltonian parameters derived according to Eq. 1 using EasySpin software package[4] for the simulation of CW ESR spectra of MOFs in the presence/absence of fullerene measured at 40 K. 15

S2 The analysis of porphyrin ring planar symmetry parameters in various MOFs according to Ref. 5. The following definitions are used: $f = g_{\parallel}/A_{\parallel}^{Cu}$, $A_{iso}^{Cu} = (A_{\parallel}^{Cu} + 2A_{\perp}^{Cu})/3$, $g_{iso} = (g_{\parallel} + 2g_{\perp})/3$. 15

S3 The best-fit parameter table provided by *Mathematica 10.0* software to the experimental spin-lattice relaxation rate data according to Eq. S1 with a numerical evaluation of the transport integral for different MOF samples. 17

S1 Experimental

S1.1 Synthesis

Materials and Methods
All solvents and reagents obtained from commercial sources were used without further purification. Pyrrole, methyl 4-formylbenzoate, p-nitrobenzaldehyde, propionic acid, pyridine and acetic anhydride were purchased from Merck. Copper(II) chloride dihydrate $Cu(Cl)_2x2H_2O$ was supplied from Fluka. Methanol (MeOH), Chloroform (CH_3Cl) and dimethylformamide (DMF) were purchased from Kemika. Diethylformamide (DEF) and triethylamine (TEA) were supplied from Sigma-Aldrich. Tetrakis(4-carboxyphenyl)porphyrin (H_2TCPP) was purchased from TCI.

Zr_{12}-acetate clusters, ($[Zr_6O_4(OH)_4(CH_3COO)_{12}]_2$) and Zr_6-methacrylate cluster ($Zr_6O_4(OH)_4$-$(C_2H_3CO_2)_{12}$) were synthesized according to the literature methods.[6,7]

5,10,15,20-Tetrakis(4-methoxycarbonylphenyl)porphyrin (TPPCOOMe) was prepared according to the literature.[8] ^{1}H NMR and PXRD data were in accordance with previously published for these compounds.

Cu(II) meso-tetra(4-carboxyphenyl)porphine (CuTCPP) was synthesized according to the literature methods.[9,10] TPPCOOMe (100 mg, 0.118 mmol) and excess $Cu(Cl)_2x2H_2O$ (250 mg, 1.47 mmol) were refluxed in DMF (20 mL) for 6h. The reaction mixture was cooled to room temperature and 20 mL of H_2O was added to the mixture. Red precipitation was formed, filtered and followed by washing three times with H_2O and dissolved in CH_3Cl, washed three times with 1M HCl, and twice with H_2O. The organic layer was dried over anhydrous magnesium sulfate and the organic solvent was evaporated to obtain the ester porphyrin complex, CuTPPCOOMe. To the THF-MeOH solution of CuTPPCOOMe, KOH (aq) (0.32 g KOH, 4 mL H_2O) was added and refluxed for 12h. After the mixture was cooled to room temperature, organic solvents were evaporated, H_2O was added to the water phase and the mixture was slightly heated until the solid was fully dissolved, then the homogeneous solution was acidified with 1 M HCl until no further precipitate was produced. The red solid was collected by filtration, washed with water and recrystallised from EtOH. PXRD and FTIR-ATR results are in good agreement with the literature data. Isolated: 60 mg (60%).

$Cu_{1.0}$-MOF-525

Zr_6-methacrylate cluster (19.55 mg, 0.012 mmol), CuTCPP (20 mg, 0.0234), NaOAc (6 mg) and 30 μL DMF were placed into a Teflon jar (14 mL) with two zirconia balls (1.6 g each). The reaction mixture was milled using IST-500 (InSolido Technologies, Croatia) mixer mill at 30 Hz for 45 min. The resulting black powder was washed with DMF (3x4 mL) and analyzed by PXRD and FTIR-ATR. Isolated: 27 mg.

$Cu_{1.0}$-PCN-223

Zr_{12}-acetate cluster (19.95 mg, 0.006 mmol) CuTCPP (20 mg, 0.0234 mmol), TEA (triethylamine, 13uL) and 40 µL MeOH (or DEF) were placed into a Teflon jar (14 mL) with two zirconia balls (1.6 g each). The reaction mixture was milled using IST-500 (InSolido Technologies, Croatia) mixer mill at 30 Hz for 90 min. The resulting black powder was washed with DMF (or DEF) (3x4 mL) and analyzed by PXRD and FTIR-ATR. Isolated: 31 mg.

$Cu_{0.1}$-MOF-525

Zr_6-methacrylate cluster (19.55 mg, 0.012 mmol) CuTCPP (2 mg, 0.0023 mmol), H_2TCPP (16.64 mg, 0.021 mmol), NaOAc (6 mg) and 30 μL DMF were placed into a Teflon jar (14 mL) with two zirconia balls (1.6 g each). The reaction mixture was milled using IST-500 (InSolido Technologies, Croatia) mixer mill at 30 Hz for 45 min. The resulting black powder was washed with DMF (3x4 mL) and analyzed by PXRD, FTIR-ATR and ICP-MS. Isolated: 24 mg. The MOF was thermally activated before subjecting to the ICP-MS analysis by exchanging the DMF with ethanol for 3 days and then heating it at 80 °C for 12 hours. FTIR data still shows traces of DMF. ICP-MS data: Zr Calculated: 18.71 Zr Found: 17.9, Cu Calculated: 0.65 Found: 0.628. Zr(IV):Cu(II) metal content determined by ICP-MS agrees with the formula C_{144} $H_{77.4}$ N_{12} O_{24} $Cu_{0.3}$ Zr_6. The discrepancies are likely due to the DMF still present in the voids of the MOF.

$Cu_{0.1}$-PCN-223

Zr_{12}-acetate cluster (19.95 mg, 0.012 mmol) CuTCPP (2 mg), H_2TCPP (16.64 mg, 0.021 mmol), TEA (13 μL) and 40 μL MeOH (or DEF) were placed into a Teflon jar (14 mL) with two zirconia balls (1.6 g each). The reaction mixture was milled using IST-500 (InSolido Technologies, Croatia) mixer mill at 30 Hz for 90 min. The resulting black powder was washed with DMF (or DEF) (3x4 mL), then with ethanol (2x3 mL), left at 80 °C for 12 hours to remove the organic solvents, and analyzed by PXRD and FTIR-ATR. Isolated: 31 mg. The elemental analysis for all samples involved in the pulse EPR measurements was conducted in the revision of the manuscript, not on the original set of materials due to them being expended in measurements. Zr-TCPP MOFs are well known for their missing-linker defects, amounting to up to forty percent without collapse of the MOF structure. Elemental analysis suggests the missing linker in all of our samples and the water in channels and clusters. The CHN results agree with the proposed formula $[Zr_6O_4(OH)_4(H_2TCPP)_{1.8}(CuTCPP)_{0.2}(OH)_4(H_2O)_7]$ C: found 49.32 (theor.49.29); H: found 3.18 (theor.3.5); N: found 4.70 (theor. 4.79).

$0.2C_{60}$@$Cu_{0.1}$-PCN-223 and $1.0C_{60}$@$Cu_{0.1}$-PCN-223

Zr_6-methacrylate cluster (19.55 mg, 0.012 mmol) CuTCPP (2 mg, 0.0023 mmol), H_2TCPP (16.64 mg, 0.021 mmol), C_{60} (3 mg), NaOAc (6 mg) and 40 μL DMF were placed into a Teflon jar (14 mL) with two zirconia balls (1.6 g each). The reaction mixture was milled using IST-500 (InSolido Technologies, Croatia) mixer mill at 30 Hz for 90 min. The resulting black powder was washed with toluene (3x5 mL) and DMF (or DEF) (2x5 mL), respectively, then with ethanol (12x3 mL), after which was left at 80 °C for 12 hours to remove the organic solvents, and analyzed by PXRD and FTIR-ATR. CHN for $0.2C_{60}$@$Cu_{0.1}$-PCN-223 fits with the proposed formula $[Zr_6O_4(OH)_4(H_2TCPP)_{1.8}(CuTCPP)_{0.2}(OH)_4(H_2O)_6(C_{60})_{0.4}]$ C: found 54.73 (theor. 55.2); H: found 3.01 (theor. 3.07); N: found 4.28 (theor. 4.26).
The same method was applied to the higher amount of C_{60} loading, $1.0C_{60}$@$Cu_{0.1}$-PCN-223, by using 16 mg of C_{60} and 50 μL DMF. Isolated: 27 mg and 36 mg, respectively for $0.2C_{60}$@$Cu_{0.1}$-PCN-223 and $1.0C_{60}$@$Cu_{0.1}$-PCN-223. ICP-MS data of $1.0C_{60}$@$Cu_{0.1}$-PCN-223 Zr Calculated: 12.53 Found: 11.84, Cu Calculated: 0.44 Found: 0.465. CHN for $1.0C_{60}$@$Cu_{0.1}$-PCN-223 agree with the formula $[Zr_6O_4(OH)_4(H_2TCPP)_{1.8}(CuTCPP)_{0.2}(OH)_4(H_2O)_6(C_{60})_{0.8}]$: C: found 58.57 (theor. 58.95); H: found 2.93 (theor. 2.87); N: found 3.70 (theor. 3.82).

$1.0C_{60}$@$Cu_{1.0}$-PCN-223

Zr_6-methacrylate cluster (19.55 mg, 0.012 mmol) CuTCPP (20 mg, 0.0234 mmol), C_{60} (16 mg), NaOAc (6 mg) and 40 µL DMF were placed into a Teflon jar (14 mL) with two zirconia balls30 (1.6 g each). The reaction mixture was milled using IST-500 (InSolido Technologies, Croatia) mixer mill at Hz for 90 min. The resulting black powder was washed with toluene (3x5 mL) and DMF (or DEF) (2x5 mL), respectively, and analyzed by PXRD and FTIR-ATR. Isolated: 37 mg. ICP-MS data of $1.0C_{60}$@$Cu_{1.0}$-PCN-223 Zr Calculated: 12.07 Found: 11.919, Cu Calculated: 4.21 Found: 4.324. Zr(IV):Cu(II) metal con- tent determined by ICP-MS agrees with the formula C_{264} H_{72} N_{12} O_{24} $Cu_3$3 Zr_6.

S1.2 Powder X-ray diffraction

PXRD data was obtained using the Panalytical Aeris Research tabletop diffractometer, with CuK radiation (40 kV and 7.5 mA) in the Bragg Brentano geometry.

S1.3 Fourier-transform infrared spectroscopy (FTIR)

Fourier-transform infrared attenuated total reflectance spectroscopy (FTIR-ATR) was conducted by the Perkin-Elmer SpectrumTwo spectrometer with a diamond cell. The spectra were collected in the range of 4400 cm^{-1} to 500 cm^{-1} , resolution of 4 cm^{-1}. Spectrum software (version 10.5.3.738, 2016) was used for data processing.

S1.4 Inductively Coupled Plasma Mass Spectrometry (ICP-MS)

ICP-MS was performed at the University of Zagreb on a Plasma ICP-MS (7900, Agilent Technologies, Singapore). Since we were limited with the revision time needed for the experiment, we were forced to give a $Cu_{1.0}$-MOF-525 polymorph instead of the $Cu_{1.0}$-PCN-223 because we were not able to prepare a phase-pure PCN-223 in this narrow time range. The two materials are polymorphs, therefore the ratio of the metals inside (Zr/Cu) is the same for both phases.

S1.5 Elemental analysis

The C, H, and N content was obtained using a Perkin Elmer 2400 Series II CHNS analyzer in an oxygen stream.

S1.6 TEM microscopy

Distribution of elements on the investigated samples were performed with energy dispersive X-ray spectroscopy (EDAX) using Jeol Centurio wide-area Silicon Drift Detector (SDD) system attached on a 200 kV cold field-emission gun (FEG) Cs-probe corrected transmission electron microscope (Jeol ARM 200 CF).

S1.7 Modeling

DFT. PCN-223 and C_{60}@PCN-223 systems were assessed at the DFT/PBE-D3 level of theory using the VASP package[11] and projector augmented-wave method as implemented in the v5.5.4 code and the standard pseudopotentials provided along with the package.[12,13] The geometries were optimized in the G-point down to the maximal residual forces of 10^{-4} eV/Å.

DFTB. Classical MD employs the DFTB+ package[14] version 19.1 with initial geometries and in the unit cell as predicted by DFT theory. The systems were parameterized using MIO force fields with missing Zr parameters substituted by chemically closely related Ti and analyzed in-house python scripts.[15,16]The MD algorithm is driven by NVT (canonical ensemble, with Nose–Hoover thermostat) equation with $T = 300$ K. The overall trajectory for the C_{60}@PCN-223 system was integrated for 100 ps with an integration time step of 1 fs.

S1.8 Solid-state nuclear magnetic resonance (NMR)

^{1}H and ^{13}C MAS (Magic-Angle Spinning), ^{1}H-^{13}C CPMAS (Cross-Polarization Magic-Angle Spinning) and HETCOR (Heteronuclear Correlation) NMR spectra were recorded on a narrow-bore 600 MHz Varian VNMRS spectrometer equipped with a 3.2 mm Varian CPMAS probe operating at ^{1}H Larmor frequency of 599.37 MHz and ^{13}C Larmor frequency of 150.71 MHz. Sample rotation frequency was 20 kHz. In one-dimensional (1D) NMR experiments the number of scans was 32 for ^{1}H MAS, 1500 for ^{13}C MAS measurements, and 1500 for ^{1}H-^{13}C CPMAS measurements while the repetition delays between scans were 1 s, 120 s, and 0.6 s, respectively. ^{1}H and ^{13}C MAS NMR spectra were measured using the Hahn-echo sequence with the echo delay of one rotation period. The ^{1}H and ^{13}C 90° pulses were 2.6 µs and 2.35 µs, respectively. ^{1}H-^{13}C CPMAS experiment employed RAMP[17] on proton channel during 1.0 ms CP block and high-power XiX proton decoupling[18,19] during acquisition. An additional CPMAS experiment was employed with a 50 µs CP block to elucidate the protonated ^{13}C signals. Two-dimensional (2D) ^{1}H-^{13}C HETCOR experiment was performed by first exciting the protons with a single pulse and letting the proton magnetization evolve in the absence of homo-/hetero-nuclear decoupling. In the next step the magnetization was transferred to carbon nuclei by RAMP CP block of 3.0 ms. 2D experiment was performed in a hypercomplex mode,[20] the number of increments along the indirectly detected dimension was 40 and 5000 scans per increment were accumulated. Tetramethylsilane was used as an external reference for both ^{1}H and ^{13}C frequency axes. The experiments were performed at 300 K.

S1.9 ESR spectroscopy

Continuous-wave (CW) and pulsed ESR experiments were performed using X-band (around 9.7 GHz) FT/CW Bruker ELEXSYS E580 spectrometer equipped with an Oxford Instruments temperature unit. CW ESR spectroscopy was performed applying modulation amplitude of 0.1 mT at 100 kHz modulation frequency from room down to liquid helium temperature.

Samples exhibiting 100% copper (II) concentration were analyzed only by CW ESR as the quantum coherence was too short to be detected due to the electron-electron spin dipolar interactions. For the pulsed ESR measurements the copper (II) concentration had to be lowered to 10%.

Magnetization decay rates were followed in the temperature interval from 5–80 K using $\pi/2$ pulse duration of 16 ns. Two-pulse echo detected field-swept ESR spectra of MOFs at $T = 80$ K were recorded in order to define spectral positions for the relaxation rate measurements. Spin-lattice relaxation times, T_1, were studied using inversion recovery (IR) pulse sequence with echo detection sequence, $\pi - t - \pi/2 - \tau - \pi - echo$, where $\tau = 100$ ns and variable t starting from the initial value of 400 ns.[21] T_1 was determined from a fit of the echo recovery amplitude to the mono ($T > 40$ K) and biexponential function ($T < 40$ K) to account for the spectral diffusion affecting IR sequence wherein the longer component was considered an approximation for T_1 .[22] The phase memory time, T_m, was derived using two-pulse electron-spin-echo decay sequence, $\pi/2 - \tau - \pi - \tau - echo$, with $\tau = 100$ ns and shots repetition time adjusted to accommodate complete spin-lattice relaxation. T_m was estimated by fitting the echo signal to the mono-exponential decay function.[21]

HYSCORE (Hyperfine Sublevel Correlation) experiment was performed using the sequence $\pi/2 - \tau - \pi/2 - t_1 - \pi - t_2 - \pi/2 - \tau - echo$, $\tau = 100$ ns and the starting values of $t_1 = t_2 = 200$ ns incremented by 16 ns in 150 steps.[21]

Measurements of echo-detected Rabi oscillations were performed with the pulse sequence $t_p - T - \pi/2 - \tau - \pi - \tau - echo$, where the nutation pulse length t_p was varied, τ was 100 ns and T was 1000 ns. In specific, the time duration of the nutation pulse, t_p, was incremented in such a way that its initial length (12 ns – 28 ns), δt, was adjusted by choosing the proper microwave power attenuation (1 dB – 8 dB) to account for maximal spin-echo signal ($\beta = \pi/2$)[21] and the field strength calculated from δt and β.[2,3]

S2 Results and discussion

S2.1 EDAX-TEM microscopy

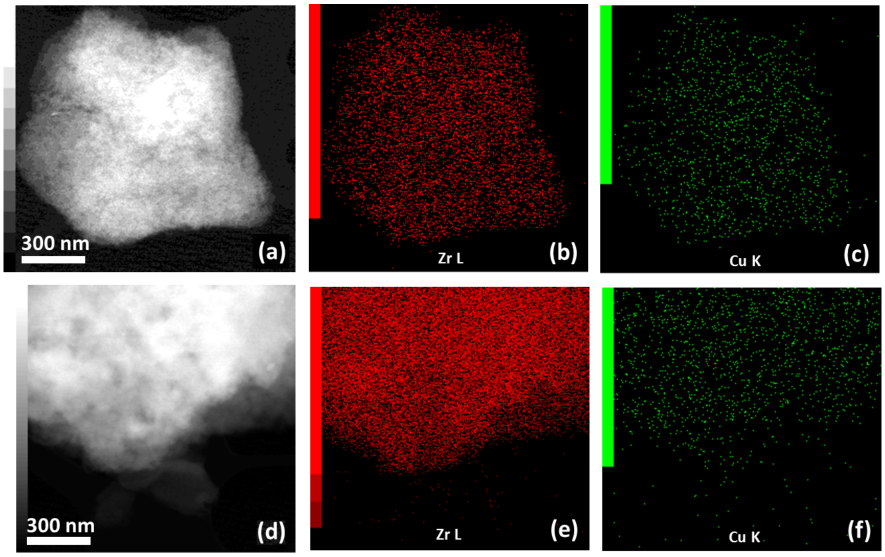

Figure S1: Elemental mapping on the selected particles of the investigated materials - (a) C_{60}@$Cu_{0.1}$-PCN-223 sample with the corresponding distribution of (b) Zr and (c) Cu; (d) C_{60}@$Cu_{1.0}$-PCN-223 sample with the corresponding distribution of (e) Zr and (f) Cu. Estimated Zr/Cu molar ratios are 45 (33 wt. Zr and 0.5 wt. Cu) and 4.0 (17 wt. Zr and 3 wt.Cu) for C_{60}@$Cu_{0.1}$-PCN-223 and C_{60}@$Cu_{1.0}$-PCN-223 samples respectively.

S2.2 DFT

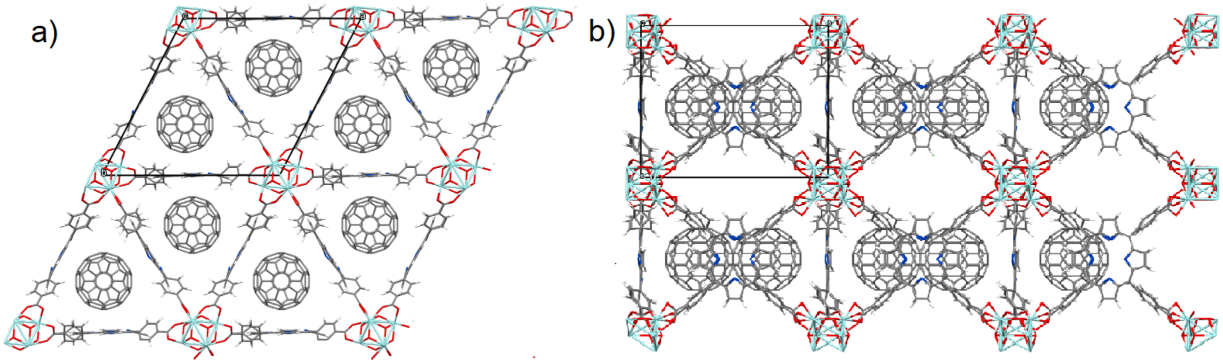

Figure S2: The view down the crystallographic a) *c*-axis and b) *a*-axis of the optimized structure of 1.0C_{60}@-PCN-223 showing the narrower channels suitable for the accommodation of DMF molecules.

S2.3 Fourier-transform infrared spectroscopy (FTIR)

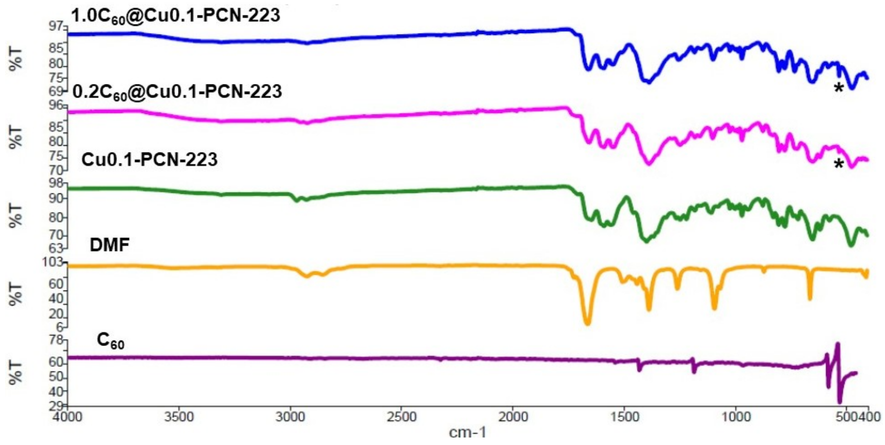

Figure S3: FTIR-ATR spectroscopy data for 1.0C_{60}@$Cu_{0.1}$-PCN-223, 0.2C_{60}@$Cu_{0.1}$-PCN-223, $Cu_{0.1}$-PCN-223, DMF, and C_{60}, respectively. The bands denoting the successful encapsulation of C_{60} are marked with an asterisk (*). The spectral distortion visible in the spectrum of the C_{60} can be ascribed to the Christiansen effect.[1] It is present in all the C_{60} spectra taken, no matter the mode of collection (ATR or transmission in a KBr pastille).

S2.4 Solid-state CPMAS and CP-HETCOR NMR spectroscopy

The ^{1}H-^{13}C CPMAS spectra of $Cu_{0.1}$-PCN-223 and C_{60}@$Cu_{0.1}$-PCN-223 are presented in Fig. S4. The assignment of ^{13}C peaks is supported by the analysis of CP–HETCOR spectrum of C_{60}@$Cu_{0.1}$-PCN-223 sample (Fig. S5). It can be noticed that the signals of DMF/DEF solvent are present in the NMR spectra of both samples. The ^{13}C signal of C_{60} at ca. 138 ppm exhibits a narrow line indicative of fast motion of the fullerene in the MOF cavity.

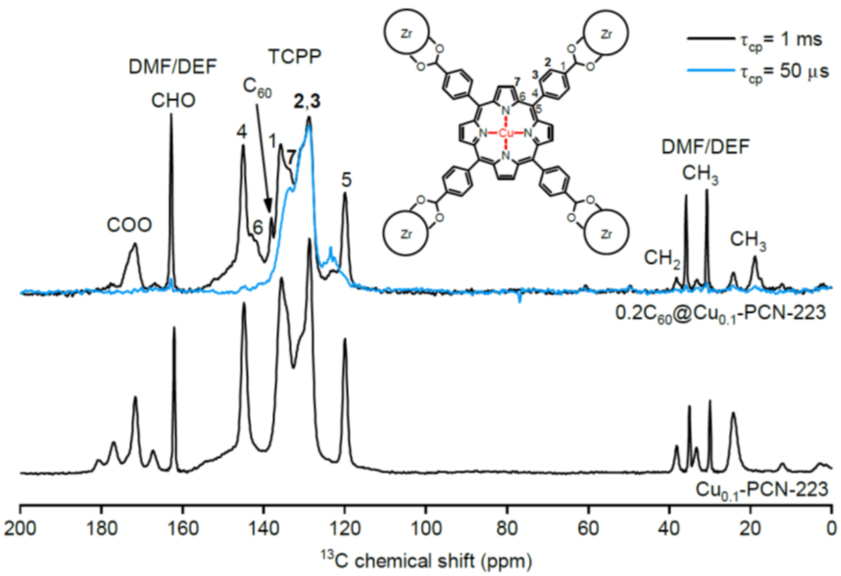

Figure S4: The ^{1}H–^{13}C CPMAS NMR spectra recorded for $Cu_{0.1}$-PCN-223 and $1.0C_{60}$@$Cu_{0.1}$-PCN-223 samples at 300 K. The blue color represents the CPMAS spectrum recorded at a very short mixing time, τ_{cp}, of 50 μs which reveals the protonated ^{13}C signals of TCPP. The ^{13}C signal of C_{60} at ca. 138 ppm is indicated by the arrow. The TCPP peaks indicated by numbers follow the notation of ^{1}H–^{13}C CP-HETCOR experiments shown in Fig. S5.

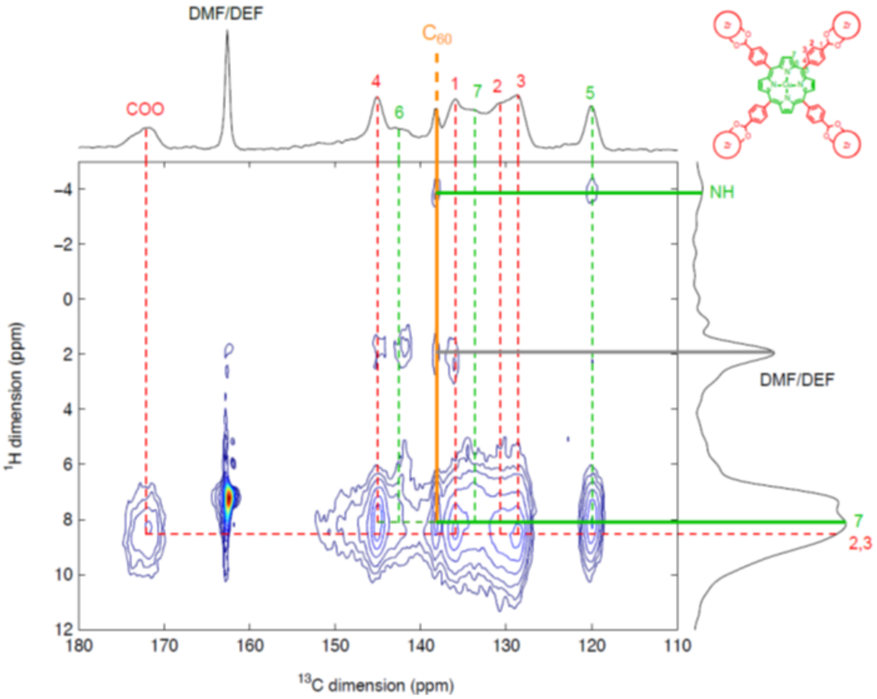

Figure S5: The ^{1}H–^{13}C CP-HETCOR NMR spectrum of $1.0C_{60}@Cu_{0.1}$-PCN-223 sample recorded at 300 K. The ^{13}C signal of C_{60} at ca. 138 ppm is indicated in the figure.The TCPP peaks indicated by numbers follow the notation of ^{1}H–^{13}C CPMAS experiments shown in Fig. S4.

S2.5 CW ESR spectroscopy

Peak-to-peak linewidth W_{pp} and g-value of fullerene-radical (FR) in $0.2C_{60}@Cu_{0.1}$-PCN-223 sample show no significant change with temperature, as can be seen in Fig. S6.

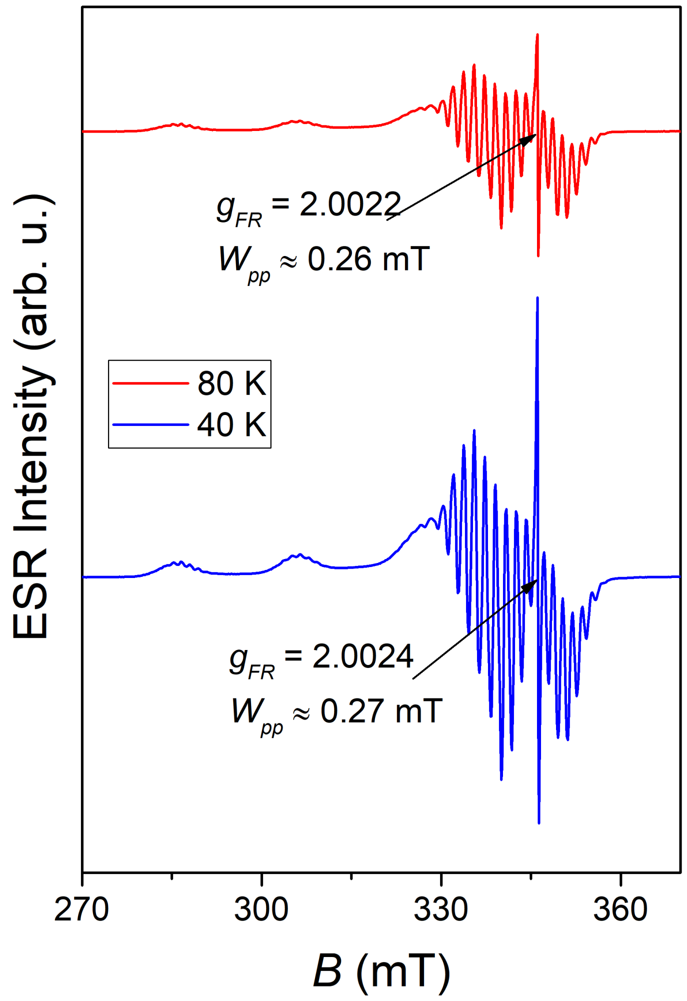

Figure S6: CW-ESR spectra of $0.2C_{60}@Cu_{0.1}$-PCN-223 at indicated temperatures. Peak-to-peak linewidth W_{pp} and g_{FR} of fullerene-radical (FR) due to the defects in fullerene structure are given.

Experimental CW ESR spectra were simulated using EasySpin software package[4] and the obtained parameters are given in Table S1.

The analysis of porphyrin ring planar symmetry in various MOFs is presented in Table S2 according to Ref. 5. The tetrahedral distortion is checked by evaluating the ratio $f = g_{\|}/A_{\|}^{Cu}$ and isotropic A_{iso} and g_{iso} tensor values. The f value in the range (110–120) cm, A_{iso}^{Cu} = (75–90)10^{-4} cm^{-1} and g_{iso} = 2.06–2.11 are indicative for the copper complex with planar symmetry.[5]

Table S1: Spin Hamiltonian parameters derived according to Eq. 1 using EasySpin software package[4] for the simulation of CW ESR spectra of MOFs in the presence/absence of fullerene measured at 40 K.

Compound Parameter	$Cu_{0.1}$-MOF-525	$Cu_{0.1}$-PCN-223	$1.0C_{60}$@ $Cu_{1.0}$-PCN-223	$0.2C_{60}$@ $Cu_{0.1}$-PCN-223	$1.0C_{60}$@ $Cu_{0.1}$-PCN-223
$g_{\parallel}$	$2.19\underline{4}$	$2.19\underline{9}$	$2.19\underline{3}$	$2.19\underline{3}$	$2.19\underline{3}$
$g_{\perp}$	$2.04\underline{7}$	$2.05\underline{0}$	$2.049\underline{5}$	$2.049\underline{5}$	$2.049\underline{5}$
$A_{\parallel}^{Cu}$, MHz	$59\underline{3}$	$60\underline{7}$	$60\underline{2}$	$59\underline{5}$	$58\underline{9}$
$A_{\perp}^{Cu}$, MHz	$6\underline{1}$	$3\underline{8}$	$6\underline{0}$	$5\underline{9}$	$5\underline{9}$
$A_{\parallel}^{N}$, MHz	$3\underline{4}$	$4\underline{7}$	-	$3\underline{8}$	$3\underline{8}$
$A_{\perp}^{N}$, MHz	$5\underline{4}$	$5\underline{4}$	-	$48.\underline{5}$	$48.\underline{5}$
Γ_L*, mT	$0.6\underline{5}$	$0.9\underline{0}$	$6.\underline{5}$	$0.7\underline{0}$	$0.7\underline{0}$
g_{FR}	-	-	$2.002\underline{4}$	$2.002\underline{5}$	$2.002\underline{5}$
W*, mT	-	-	$0.3\underline{0}$	$0.3\underline{0}$	$0.3\underline{0}$

* Lorentzian linewidth of the CW ESR spectra.
The underlined digit represents the uncertainty of the given decimal position within the respective decade.

Table S2: The analysis of porphyrin ring planar symmetry parameters in various MOFs according to Ref. 5. The following definitions are used: $f = g_{\parallel}/A_{\parallel}^{Cu}$, $A_{iso}^{Cu} = (A_{\parallel}^{Cu} + 2A_{\perp}^{Cu})/3$, $g_{iso} = (g_{\parallel} + 2g_{\perp})/3$.

Compound	$g_{\parallel}$	$g_{\perp}$	$A_{\parallel}^{Cu*}$	$A_{\perp}^{Cu*}$	g_{iso}	A_{iso}^{Cu*}	f
$Cu_{0.1}$MOF-525	2.194	2.047	198.0	20.4	2.096	79.6	110.8
$Cu_{0.1}$-PCN-223	2.199	2.05	202.7	12.7	2.100	76.0	108.5
$1.0C_{60}$@$Cu_{0.1}$-PCN-223	2.193	2.0495	196.7	19.7	2.097	78.7	111.5

* hyperfine coupling constants are in cm^{-1} and multiplied by 10^4.

S2.6 Pulse ESR spectroscopy

The spin-lattice relaxation rate experimental data, $1/T_1$, were analysed according to[23,24]

$$1/T_1 = aT + b\frac{T^9}{\theta^7}J_8 + c\frac{e^{\Delta/T}}{(e^{\Delta/T}-1)^2}, \tag{S1}$$

where the first term denotes a direct one-phonon process while the second and third terms represent a two-phonon Raman and local-mode contribution processes, respectively. The best-fit parameters are given in Table 1 and with more statistical details in Table S3 while theoretical curves are presented in Fig. S7. The contribution of individual components i.e. direct, Raman and local modes are demonstrated in Fig. S8 referring to $Cu_{0.1}$-PCN-223.

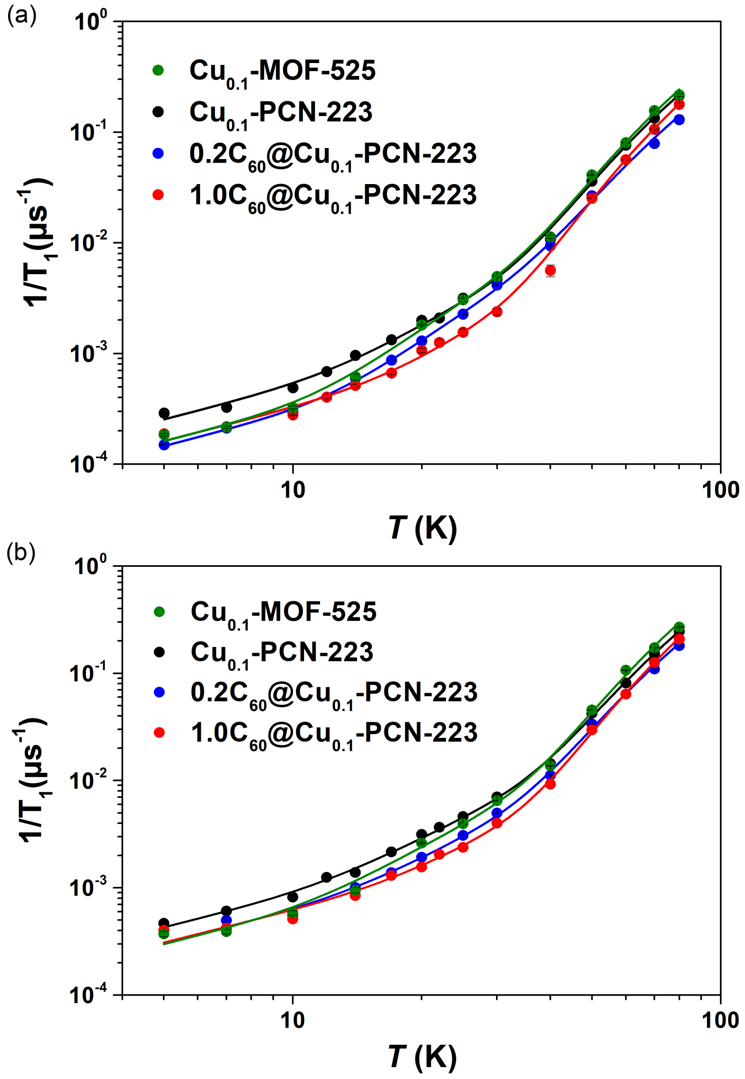

Figure S7: Temperature dependence of the electron spin-lattice relaxation rate, $1/T_1$, in various MOFs as indicated in the legend for two spectral positions: a) $g_{\parallel}$ and b) $g_{\perp}$. Full lines denote simulations of the experimental data according to the Eq. S1 with the parameters given in Table 1.

Table S3: The best-fit parameter table provided by *Mathematica 10.0* software to the experimental spin-lattice relaxation rate data according to Eq. S1 with a numerical evaluation of the transport integral for different MOF samples.

$Cu_{0.1}$-MOF-525 at $g_\perp$	Estimate	Asymp. SE	TStat	PValue
a, $1/(K\mu s)$	0.0000595148	0.0000114247	5.2093	0.00124027
b, $1/(K^2\mu s)$	0.0000405516	0.0000163535	2.47969	0.0422294
θ, K	65.7681	34.6542	1.89784	0.0995213
c, $1/\mu s$	9.37904	4.15521	2.25718	0.0585749
Δ, K	292.055	30.3373	9.62693	0.000027473

$Cu_{0.1}$-MOF-525 at $g_\parallel$	Estimate	Asymp. SE	TStat	PValue
a, $1/(K\mu s)$	0.0000322479	3.10918895931045*^-6	10.3718	0.0000168097
b, $1/(K^2\mu s)$	0.0000416121	0.0000151122	2.75354	0.0283589
θ, K	76.7107	21.8924	3.50399	0.00993945
c, $1/\mu s$	6.80322	3.49001	1.94934	0.092257
Δ, K	284.76	34.6752	8.21221	0.0000770885

$Cu_{0.1}$-PCN-223 at $g_\perp$	Estimate	Asymp. SE	TStat	PValue
a, $1/(K\mu s)$	0.0000403209	9.522322147713558*^-6	4.23435	0.00173182
b, $1/(K^2\mu s)$	0.000085535	6.945061125215895*^-6	12.3159	2.2871313944293284*^-7
θ, K	66.5662	18.9043	3.52122	0.00552715
c, $1/\mu s$	7.3275	2.1178	3.45995	0.00612322
Δ, K	289.795	20.4161	14.1944	5.9358785043992486*^-8

$Cu_{0.1}$-PCN-223 at $g_\parallel$	Estimate	Asymp. SE	TStat	PValue
a, $1/(K\mu s)$	0.0000507359	2.602288420144728*^-6	19.4966	2.7530106388231864*^-9
b, $1/(K^2\mu s)$	0.0000309706	0.0000108445	2.85588	0.017077
θ, K	74.1442	18.3317	4.04459	0.00234388
c, $1/\mu s$	5.35278	2.07279	2.58241	0.0273033
Δ, K	273.174	25.8003	10.588	9.392630590415846*^-7

$0.2C_{60}$@$Cu_{0.1}$-PCN-223 at $g_\perp$	Estimate	Asymp. SE	TStat	PValue
a, $1/(K\mu s)$	0.0000613378	9.08595953951045*^-6	6.75083	0.000515159
b, $1/(K^2\mu s)$	0.0000283821	0.0000135929	2.08801	0.0818226
θ, K	77.0817	36.3691	2.11943	0.0783454
c, $1/\mu s$	4.98247	2.38626	2.08798	0.0818265
Δ, K	279.667	32.5421	8.594	0.000136459

$0.2C_{60}$@$Cu_{0.1}$-PCN-223 at $g_\parallel$	Estimate	Asymp. SE	TStat	PValue
a, $1/(K\mu s)$	0.0000292037	1.7247552553761394*^-6	16.9321	6.140500743439979*^-7
b, $1/(K^2\mu s)$	0.0000332148	7.322044086507023*^-6	4.53627	0.00267959
θ, K	81.7474	11.9918	6.81697	0.000249336
c, $1/\mu s$	3.49049	2.14316	1.62867	0.147407
Δ, K	280.914	36.2472	7.74994	0.000111575

$1.0C_{60}$@$Cu_{0.1}$-PCN-223 at $g_\perp$	Estimate	Asymp. SE	TStat	PValue
a, $1/(K\mu s)$	0.0000617872	5.39903901154222*^-6	11.4441	1.1521238367174776*^-6
b, $1/(K^2\mu s)$	0.0000229304	0.0000150046	1.52823	0.160807
θ, K	94.0135	44.0175	2.13582	0.0614375
c, $1/\mu s$	8.34757	3.18672	2.61949	0.0278343
Δ, K	306.208	28.2415	10.8425	1.8179861900868364*^-6

$1.0C_{60}$@$Cu_{0.1}$-PCN-223 at $g_\parallel$	Estimate	Asymp. SE	TStat	PValue
a, $1/(K\mu s)$	0.0000323843	1.761378966043931*^-6	18.3858	4.878041621487287*^-9
b, $1/(K^2\mu s)$	0.0000175837	0.0000140868	1.24824	0.240377
θ, K	93.6518	39.953	2.34405	0.041055
c, $1/\mu s$	5.71382	2.41711	2.3639	0.039688
Δ, K	289.005	29.4901	9.80006	1.9122023678210543*^-6

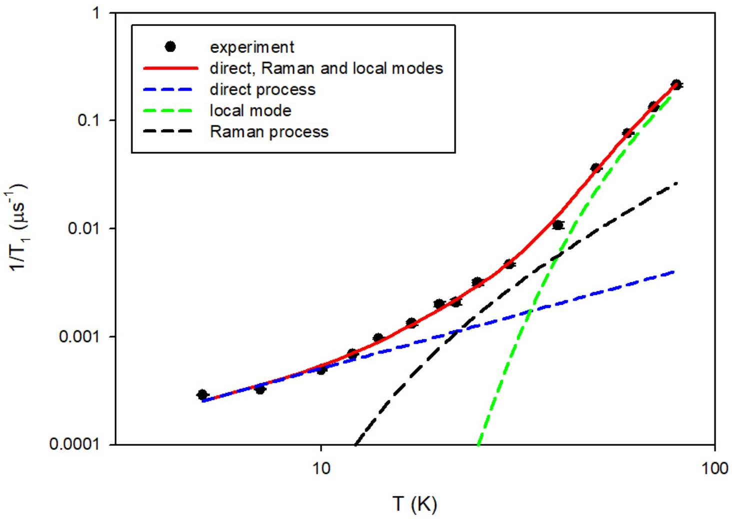

Figure S8: Temperature dependence of the electron spin-lattice relaxation rate, $1/T_1$, in $Cu_{0.1}$-PCN-223 at $g_{||}$ spectral position. Experimental data (symbols) were simulated (lines) according to the Eq. S1 with the parameters given in Table 1. Individual components of direct, Raman and local modes are presented as indicated in the legend.

The temperature dependence of phase-memory relaxation rate is presented in Fig. S9. Below 40 K there is no thermally activated processes while in the temperature interval from 40–80 K a thermally activated behavior can be noticed[25] with the activation energies increase in the presence of fullerene.

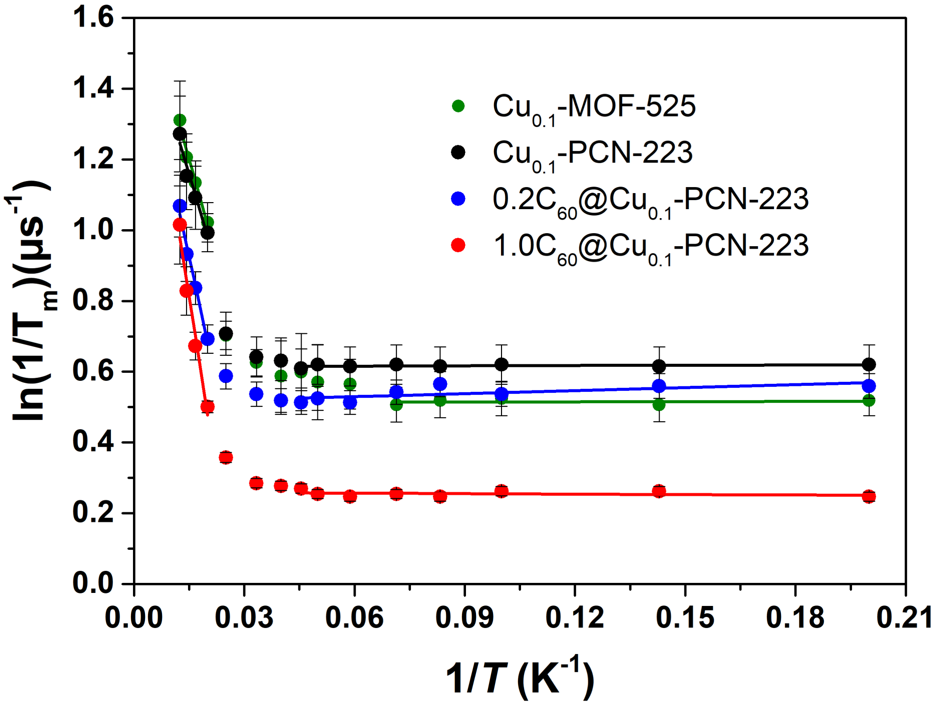

Figure S9: Temperature dependence of the electron spin phase-memory relaxation rate, $1/T_m$, in various MOFs at $g_{||}$ spectral position. The best fits according to Arrhenius equation are indicated with full lines. In the temperature interval from 40–80 K the activation energies are: 35±5 K ($Cu_{0.1}$-PCN-223, black), 48±5 K ($0.2C_{60}$@$Cu_{0.1}$-PCN-223, blue), 67±8 K ($1.0C_{60}$@$Cu_{0.1}$-PCN-223, red) and 37±4 K ($Cu_{0.1}$-MOF-525, green).

Hahn echo decay curves showing strong modulation pattern are presented in Fig. S10.

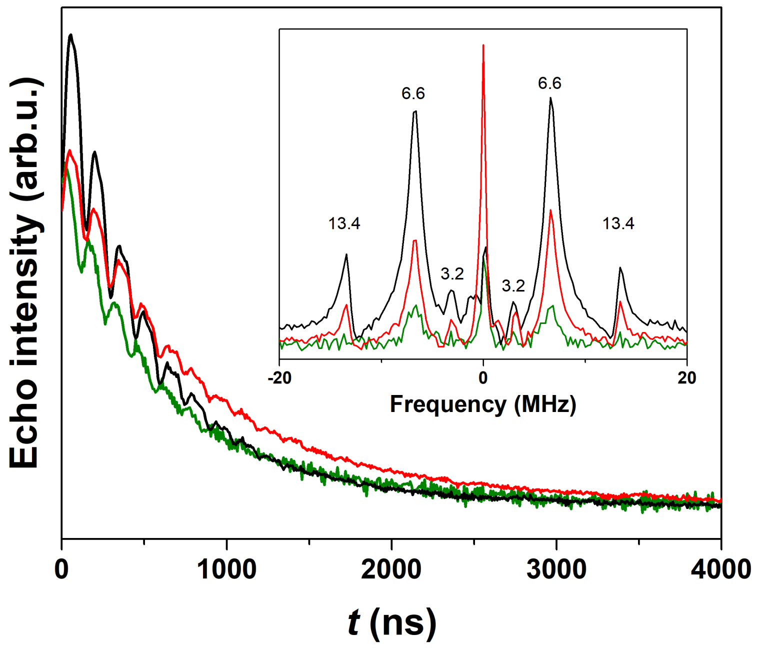

Figure S10: Hahn echo intensity decay curves detected at 5 K and $g_{\|}$ spectral position for $Cu_{0.1}$-PCN-223 (black), $1.0C_{60}$@$Cu_{0.1}$-PCN-223 (red) and $Cu_{0.1}$-MOF-525 (green). In the Inset to the Figure the Fourier transform of the time domain data is presented.

Rabi oscillations detected at $g_\perp$ spectral position are presented in Fig. S11.

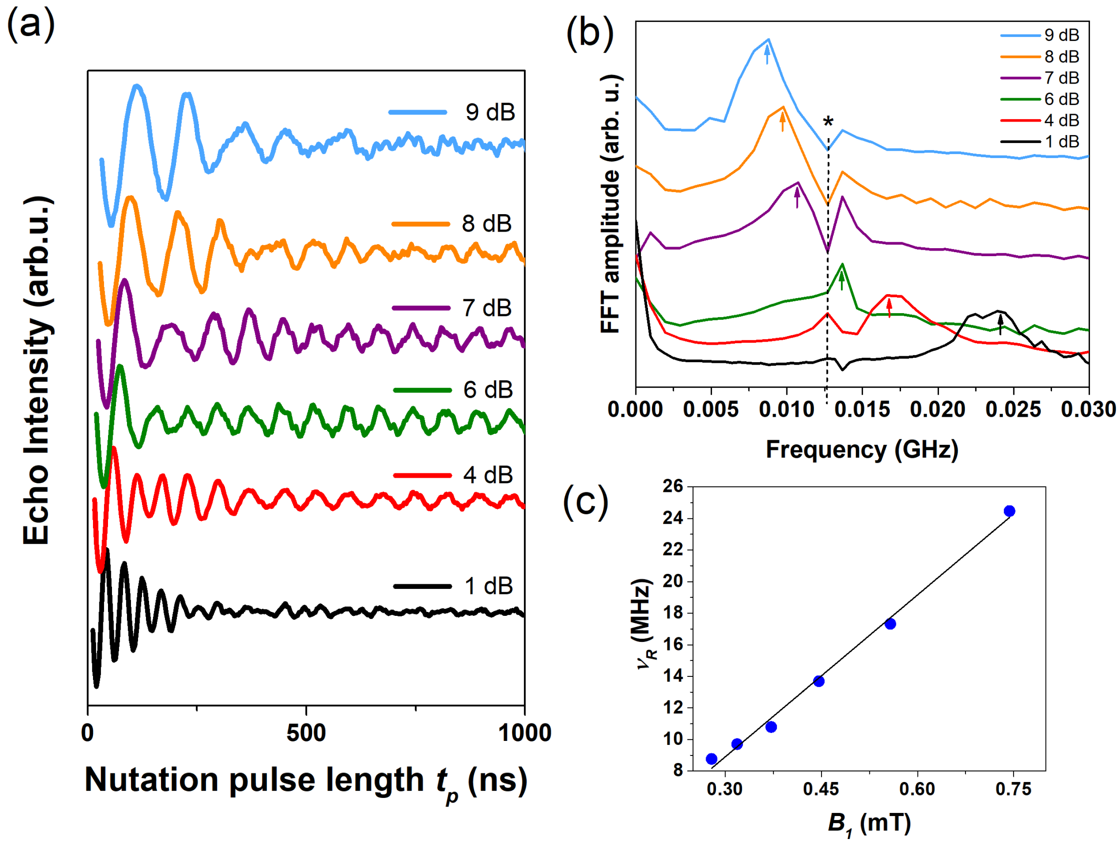

Figure S11: (a) Rabi oscillations recorded for $Cu_{0.1}$-PCN-223 at 20 K and $g_\perp$ spectral position applying various microwave power attenuation. (b) Fast Fourier transform (FFT) of the Rabi oscillations with an asterisk denoting Larmor frequency of proton spins ≈13.5 MHz while arrows denote Rabi frequencies. (c) Linear dependence of the Rabi frequency, ν_R, as a function of the oscillating microwave field, B_1, calculated according to refs. 2 and 3.

The contribution of different C atoms to superhyperfine interaction could be seen in Fig. S12 where 8 different Cu–C distances are indicated.

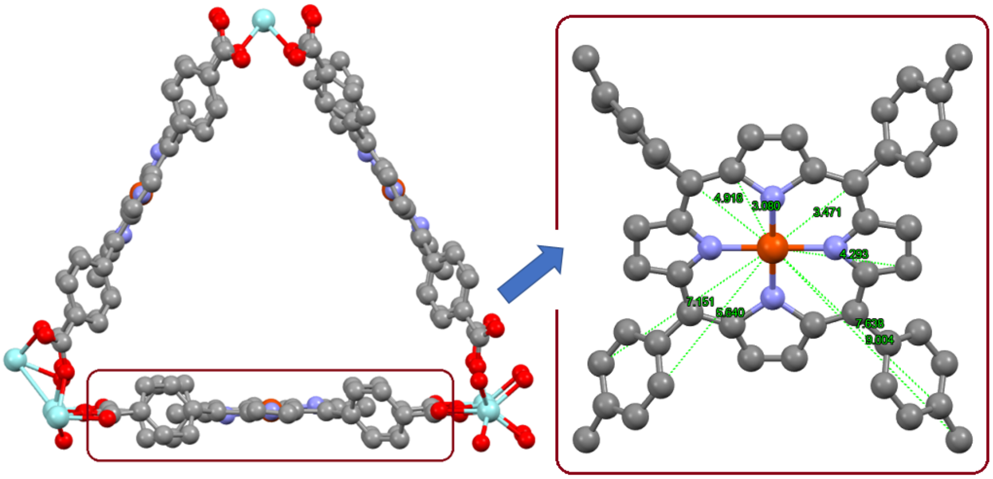

Figure S12: Left: Asymmetric unit of $Cu_{1.0}$-PCN-223 (Zr-light blue, N-blue, O-red, C-gray, Cu-orange). Right: 1/3 part of the asymmetric unit with 8 different Cu–C distances indicated.

References

[1] N. Biliškov, *Phys. Chem. Chem. Phys.*, 2022, **24**, 19073–19120.

[2] G. R. Eaton, S. S. Eaton, R. W. Quine, D. Mitchell, V. Kathirvelu and R. T. Weber, *J. Magn. Reson.*, 2010, **205**, 109–113.

[3] G. Jeschke, in *Instrumentation and Experimental Setup*, Springer US, Boston, MA, 2007, pp. 17–47.

[4] S. Stoll and A. Schweiger, *J. Magn. Reson.*, 2006, **178**, 42–55.

[5] V. Gómez-Vidales, A. Borja-Miranda, S. Cortez-Maya, O. Amelines-Sarria, M. Rivera and M. Martínez-García, *Open Chem. J.*, 2016, **3**, 25–34.

[6] M. Puchberger, F. R. Kogler, M. Jupa, S. Gross, H. Fric, G. Kickelbick and U. Schubert, *Eur. J. Inorg. Chem.*, 2006, 3283–3293.

[7] G. Kickelbick and U. Schubert, *Chcm. Ber. Recueil*, 1997, **130**, 473–477.

[8] K. Lu, C. He, N. Guo, C. Chan, K. Ni, R. R. Weichselbaum and W. Lin, *Journal of the American Chemical Society*, 2016, **138**, 12502–12510.

[9] D. Feng, Z.-Y. Gu, J.-R. Li, H.-L. Jiang, Z. Wei and H.-C. Zhou, *Angew. Chem. Int. Ed.*, 2012, **51**, 1–5.

[10] D. Feng, H.-L. Jiang, Y.-P. Chen, Z.-Y. Gu, Z. Wei and H.-C. Zhou, *Inorg. Chem.*, 2013, **52**, 12661—-12667.

[11] G. Kresse and J. Furthmüller, *Physical review B*, 1996, **54**, 11169.

[12] G. Kresse and D. Joubert, *Physical review b*, 1999, **59**, 1758.

[13] J. P. Perdew, M. Ernzerhof and K. Burke, *The Journal of chemical physics*, 1996, **105**, 9982–9985.

[14] B. Hourahine, B. Aradi, V. Blum, F. Bonafé, A. Buccheri, C. Camacho, C. Cevallos, M. Deshaye, T. Dumitrică, A. Dominguez *et al.*, *The Journal of chemical physics*, 2020, **152**, 124101.

[15] M. Elstner, D. Porezag, G. Jungnickel, J. Elsner, M. Haugk, T. Frauenheim, S. Suhai and G. Seifert, *Phys. Rev. B*, 1998, **58**, 7260–7268.

[16] G. Dolgonos, B. Aradi, N. H. Moreira and T. Frauenheim, *Journal of chemical theory and computation*, 2010, **6**, 266–278.

[17] G. Metz, X. L. Wu and S. O. Smith, *J. Magn. Reson. A*, 1994, **110**, 219–227.

[18] A. E. Bennett, C. M. Rienstra, M. Auger, K. V. Lakshmi and R. G. Griffin, *J. Chem. Phys.*, 1995, **103**, 6951–6958.

[19] A. Detken, E. H. Hardy, M. Ernst and B. H. Meier, *Chem. Phys. Lett.*, 2002, **356**, 298–304.

[20] D. J. States, R. A. Haberkorn and D. J. Ruben, *J. Magn. Reson.*, 1982, **48**, 286–292.

[21] A. Schweiger and G. Jeschke, *Principles of Pulse Electron Paramagnetic Resonance*, Oxford University Press, Oxford, 2001.

[22] S. S. Eaton and G. R. Eaton, *Relaxation Times of Organic Radicals and Transition Metal Ions. In: Berliner L.J., Eaton G.R., Eaton S.S. (eds) Distance Measurements in Biological Systems by EPR. Biological Magnetic Resonance, vol 19.*, Springer, Boston, MA, 2002.

[23] Y. Zhou, B. E. Bowler, G. R. Eaton and S. S. Eaton, *J. Magn. Reson.*, 1999, **139**, 165–174.

[24] M. Kveder, D. Merunka, M. Jokić, J. Makarević and B. Rakvin, *Phys. Rev. B*, 2009, **80**, 052201—052205.

[25] M. A. Augustyniak-Jabłokow, K. Tadyszak, R. Strzelczyk, R. Fedaruk and R. Carmieli, *Carbon*, 2019, **152**, 98–105.
